# Why Do You Want a Romantic Relationship? Individual Differences in Motives for Romantic Relationship Pursuit

**DOI:** 10.1177/01461672251331699

**Published:** 2025-05-12

**Authors:** Geoff MacDonald, Serena Thapar, William S. Ryan, Joanne M. Chung, Elaine Hoan, Yoobin Park

**Affiliations:** 1 University of Toronto, ON, Canada; 2 McGill University, Montreal, QC, Canada; 3 University of California, San Francisco, USA

**Keywords:** Singlehood, Self Determination Theory, Romantic Relationships, Motivation/Goal Setting

## Abstract

Relationship science currently lacks a theoretical approach to capture the variety of motivations potentially underlying the pursuit of romantic relationships. We introduce the Autonomous Motivation for Romantic Pursuit Scale (AMRPS) which conceptualizes and measures motivations ranging in levels of autonomy (from a motivation to intrinsic motivation), based on self-determination theory (SDT). In Study 1 (*N* = 1,280), we show how the motivations assessed using AMRPS relate to existing constructs implicated in romantic pursuit (e.g. fear of being single, commitment readiness), thereby organizing them into a coherent theoretical framework. In Study 2 (*N* = 3,186), we validate this approach using longitudinal data, showing singles who are higher in autonomous motivation for relationship pursuit are more likely to be partnered six months later. These studies demonstrate the usefulness of SDT for consolidating into one theoretical and measurement framework the variety of motivations (including an absence of motivation) for pursuing romantic relationships.

## Introduction

Arguably, a fundamental assumption running through relationship science is that a vast majority of people desire a romantic relationship. For example, evolutionary perspectives suggest that desires for sex (e.g. Schmitt, 2015), belongingness (e.g. Baumeister & Leary, 1995), and emotional attachment (e.g. Simpson & Belsky, 2008) should be universal, and data suggest that romantic partnerships are the relationship through which many in industrialized societies seek to fulfill these evolutionarily innate desires (Finkel et al., 2015). As such, social psychologists often conceptualize motivation for dating and marital relationships as a sort of natural instinct, a force that pushes people toward partnering for the inherent gratification of evolved reward pathways.

However, differing perspectives within and outside of social psychology are increasingly arising, challenging the notion that a draw toward romantic partnership is fully natural or inevitable. For example, DePaulo and Morris (2005, 2006) have noted that there is a significant percentage of people who do not wish to be in a romantic relationship. Indeed, research has identified a substantial group of singles who appear to be primarily motivated by a desire for independence and to be relatively weakly motivated by a variety of social desires, including that for romantic partnership (Park et al., 2023). Rather than framing innate desire as the key motivator behind romantic relationship pursuit, other perspectives have identified factors such as family and social pressure that can provide motivation to pursue romantic partnering. For example, Day et al. (2011) argue that industrialized societies typically include elements of *committed relationship ideology*, or social structures that incentivize living in monogamous romantic partnerships, which lead to devaluation and discrimination against those who prefer to live outside of committed romantic relationships (Day, 2013; Day et al., 2011). These considerations highlight that a relationship science that is founded on the assumption that romantic relationships are pursued solely for their inherent rewards, while ignoring the variety of other less intrinsic reasons that may lead individuals toward romantic relationships, will be incomplete. In the present research, we use self-determination theory (SDT) to conceptualize the range of motivations that may underlie relationship pursuit as well as to develop and validate a scale designed to take this range of motivation into consideration.

### The Self-Determination Continuum of Motivation

Although the notion that a range of motivations is likely to underlie the pursuit of romantic relationships should be fairly uncontroversial, the challenge is to collate as thoroughly and systematically as possible the various motivations that may underpin such pursuit. SDT offers a singular theoretical framework for integrating, organizing, and understanding such motivations. SDT has received significant empirical support in its application to various instrumental goals, such as education (e.g. Reeve & Cheon, 2021), sports and leisure (Pelletier et al., 1995), and health behavior change (e.g. Ntoumanis et al., 2021). It has also been applied to goals in interpersonal relationships, such as childhood friendships (Richard & Schneider, 2005) and within existing adult romantic relationships (Blais et al., 1990; Shoikhedbrod et al., 2023).

SDT is a meta theory of human motivation, which suggests that all behavior has a locus of control and that variation in perceived locus of control has important implications for behavior and well-being (Deci & Ryan, 1985; Ryan & Deci, 2017). Specifically, SDT considers the extent to which one’s behaviors are autonomous (i.e. originating from within oneself) versus controlled or heteronomous (i.e. originating from intrapersonal or interpersonal pressure). SDT posits a taxonomy of motivation that organizes motives along a *continuum*, with motivations or regulatory styles that range from being controlled, or engaging in an activity due to extrinsic pressures, to being fully autonomous and pursuing an activity for its own intrinsic worth (Deci & Ryan, 2000; Ryan & Connell, 1989). More autonomous motivation is more stable over time and associated with greater goal achievement and a more positive subjective experience in pursuit of goals (e.g. Baard et al., 2004; Moller et al., 2014).

At the low autonomy end of the continuum is *amotivation*, or a lack of motivation to engage in a behavior. This is associated with an impersonal locus of control which describes a perceived lack of control or intentionality or no perceived relevance to the self (Deci & Ryan, 1985; Koestner & Levine, 2023). Next on the continuum is the first and most controlled form of motivation, *external regulation*. This regulatory style reflects engaging in behaviors out of compliance, to avoid punishment, or to obtain a reward (Deci & Ryan, 2000; Ryan & Connell, 1989). Here, the locus of control is external and behavior is regulated by factors external to both the self and the activity. Just after external regulation on the continuum is *introjected* regulation where one engages in behaviors to enhance one’s self-worth (i.e. positive introjected), or out of obligation or to avoid negative feelings, such as guilt or shame (i.e. negative introjected). With introjection, the locus of control is still somewhat external. Although the pressure is experienced as originating within the self, it is focused on obtaining evaluative, external outcomes. Next on the continuum is *identified* regulation, which reflects engaging in a behavior because one finds it personally meaningful and endorses the value of that activity.^1^ Finally, *intrinsic motivation* reflects engaging in behaviors because they are inherently satisfying. Here, the satisfaction derived from engaging in the behavior itself is what motivates continued engagement. Thus, the motivational continuum begins with amotivation, moves through more controlled forms of motivation (extrinsic and introjected), where the locus of control is external, and ends with more autonomous motivations (identified and intrinsic) where the locus of control is internal and the person experiences a sense of agency and endorsement of the behavior.

### Self-Determination Theory and Motivation for Relationships

Given SDT’s theoretically grounded model of the range of possible motivations, this may be a useful framework for understanding the variety of reasons singles may have for (not) pursuing a romantic relationship. Most SDT research in the romantic relationship domain has focused on motivations *within* romantic relationships, developing scales such as the Couple Motivation Questionnaire (Blais et al., 1990), which assesses motivations for being involved in one’s current romantic relationship, and the Motivations for Relational Activities scale, which evaluates motivations toward relational activities within romantic relationships (Gaine & La Guardia, 2009). However, these scales do not map well onto a singlehood context. For example, the amotivation item, “I don’t know; I feel helpless to the fact that sooner or later we are going to separate,” (Blais et al., 1990) does not translate well to single people who do not want to date at all. Indeed, few researchers have sought to apply SDT to understand the motivations for pursuing (or not pursuing) romantic relationships amongst single individuals. One notable exception is Gravel et al. (2016) who developed a scale for motivation to engage in sexual (but not romantic) relationships. While the work by Gravel et al. (2016) is excellent, the exclusive focus of the scale on sexual activities across casual and committed contexts does not suit the goals of the current research well. Further, translation of many of the scale’s items from a sexual to relationship context would have been challenging (e.g. the identified item, “Because I feel it’s important to experiment sexually,” does not translate well to a relationship context).

The existing literature on self-determined motivations for seeking and entering romantic relationships is limited to adolescence and young adulthood (Kindelberger & Tsao, 2014; Nakai, 2020). Among this population, the three most common reasons for relationship initiation are identity, intimacy, and social status (Zimmer-Gembeck et al., 2012). Kindelberger and Tsao (2014) developed the Romantic Motivation Scale (RMS), a 16-item scale that assesses four factors on the self-determined motivation continuum: amotivation, external regulation, identified regulation, and intrinsic regulation. The correlational structure of the scale suggested that different types of motivations were indeed associated differently with outcomes related to romantic experiences. For example, while higher identification regulation was associated with greater odds of having had sexual intercourse, higher extrinsic motivation was associated with lower odds of having had sex. However, the four factors of the RMS do not map on fully to our proposed six factor motivational structure, and the items of this scale are not suitable for adult participants (e.g. the identified item, “It’s important to discover what a boy/girl is like”). Overall, while work with adolescents has suggested the potential utility of a SDT approach to motivation to pursue a romantic relationship, a conceptual and measurement framework suitable for adult participants has not yet been developed.

### The Present Research

We developed a series of items to capture the six motivations based on existing measures of motivation. Specifically, we adapted items from the Comprehensive Relative Autonomy Index that Sheldon et al. (2017) developed based on existing SDT measures, altering the items to fit a relationship pursuit context or creating new items where direct adaptation was not feasible. Items with low item-total correlations or little variability were revised following a pilot study (*N* = 500) that tested these items. In two subsequent studies, we further examined the structure of the measure, including fitting a bifactor model to test whether it captures both the global quantity and the specific quality of motivation (Howard et al., 2018). A more detailed description of these studies is available in the Supplemental Materials.

The primary goal of the present research was to examine the utility of this SDT-informed scale of motivations for romantic relationship pursuit (Autonomous Motivation for Romantic Pursuit Scale [AMRPS]). We examined whether different types of motivations from the AMRPS were meaningfully related to and able to organize a suite of measures related to motivation to partner including general social motivations (attachment orientation, social goals, fear of being single, sociosexual orientation, communal strength) and interest in partnering (commitment readiness, desire for a partner, interest in serious/casual relationships). Then, in Study 2, we collected longitudinal data in order to examine the ability of our framework to predict which singles are (and are not) most likely to transition from singlehood to partnered status.

## Study 1

The primary aim of Study 1 was to examine the validity of the AMRPS by examining its correlations with a comprehensive set of constructs in the existing literature that tap into individual differences capturing broader interpersonal motivations potentially relating to romantic relationship pursuit. Of note, we did not have a priori hypotheses as to how each of the motivations would relate to each of the individual differences, opting instead for an “explore and replicate” approach. Two samples were sequentially collected, with Sample 1A collected first and Sample 1B collected later for replication purposes. We also included four measures capturing romantic interest and desire (commitment readiness, desire for a partner, and interest in serious and casual relationships), with expectations that they would all be correlated with but not equivalent to the motivations from AMRPS. Overall, we sought to examine whether our scale could provide a broad organizing framework for relationship motivations without being redundant with established measures.

## Methods

### Participants

#### Sample 1A

A sample of 575 participants who were at least 18 years old and were not currently involved in a romantic relationship was recruited from Prolific to participate in a survey hosted on the data collection platform Qualtrics. In all studies, the surveys were only available to those who indicated when signing up with Prolific that they were not in a romantic relationship. However, we also included a question in our survey, “Are you currently in a romantic relationship?” to which participants could answer yes or no in order to ensure participants’ single status (the survey terminated for participants who indicated “*yes*”). The survey was in English and available to any participant using Prolific globally. We excluded 75 participants who did not complete the study, failed attention checks, or who reported being dishonest in answering the questions. The final sample consisted of 500 participants (246 men, 254 women) who were 24.8 years old on average (*SD* = 8.7; range = 18–74). Participants described their ethnic/racial background as White (*n* = 357), Middle Eastern (*n* = 31), Latino/Hispanic (*n* = 18), South Asian (*n* = 13), African (*n* = 9), East Asian (*n* = 6), Caribbean (*n* = 1), multiethnic (*n* = 26; multiple responses allowed), or other (*n* = 34). Five participants provided no response. Participants were primarily residing in Poland (*n* = 120), United Kingdom (*n* = 97), Portugal (*n* = 87), and Italy (*n* = 43). Participants identified as heterosexual (*n* = 394), bisexual (*n* = 58), uncertain or questioning (*n* = 22), gay (*n* = 11), lesbian (*n* = 4), queer (*n* = 3), or other (*n* = 5); three participants chose not to answer. Most participants had previously been in a romantic relationship (*n* = 311), which ended an average of 27.8 months prior to the start of the study (*SD* = 33.3; range = 1–300).

#### Sample 1B

A sample of 1,005 participants who were at least 18 years old and were not currently involved in a romantic relationship was recruited from Prolific. We excluded 225 participants who did not complete the study, failed attention checks, or who reported being dishonest in answering the questions. The final sample consisted of 780 participants (392 men, 382 women, 5 other, 1 prefer not to answer) who were 24.7 years old on average (*SD* = 6.5; range = 18–65). Participants described their ethnic/racial background as African (258), White (*n* = 231), Latino/Hispanic (*n* = 209), Middle Eastern (*n* = 12), South Asian (*n* = 7), East Asian (*n* = 3), Caribbean (*n* = 1), multiethnic (*n* = 29; multiple responses allowed), or other (*n* = 30). Participants primarily resided in South Africa (*n* = 247), Mexico (*n* = 175), Portugal (*n* = 73), and Poland (*n* = 64). Participants identified as heterosexual (*n* = 603), bisexual (*n* = 82), uncertain or questioning (*n* = 28), gay (*n* = 26), lesbian (*n* = 9), queer (*n* = 12), or other (*n* = 17); three participants chose not to answer. Most participants had previously been in a romantic relationship (*n* = 567), which ended an average of 22.7 months prior to the start of the study (*SD* = 22.1; range = 1–204). We ensured that both samples were large enough for exploring the bifactor structure of the model (Hancock & French 2013).

### Measures: Autonomous Motivation for Romantic Pursuit

**Figure 1.**
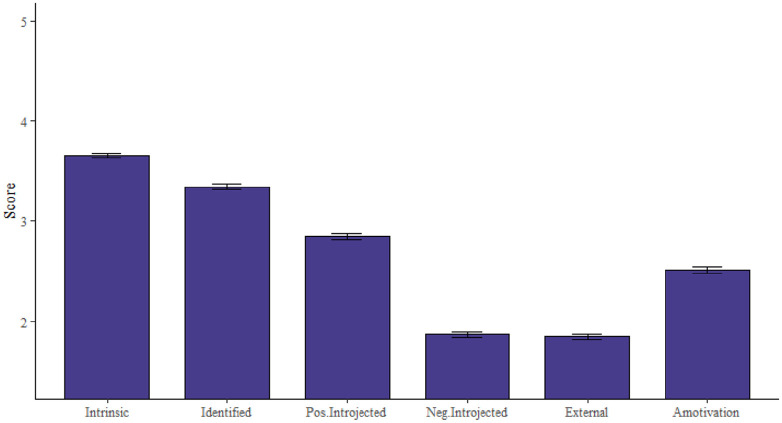
Mean endorsement of each AMRPS motivation. *Note*. AMRPS = Autonomous Motivation for Romantic Pursuit Scale

We drafted a 24-item scale of motivation for relationship pursuit based on previous SDT scale development research (Sheldon et al., 2017). Two of the current authors with expertise in both singlehood and romantic relationship research adapted each of the items (or developed a related item in cases where a direct adaptation was not possible) from the Sheldon et al. (2017) 24-item scale to suit a relationship pursuit context. Participants answered each item responding to the prompt, “To the extent that you would currently like to be in a romantic relationship, why is that?” with a scale ranging from 1 (*strongly disagree*) to 5 (*strongly agree*). Some items (that had low item to total correlations or little variability) were revised following a pilot study testing these items. The full list of items used in the pilot study can be found in the Supplemental Material (Table S1). The final 24 items used in this study are presented in Table 1. The internal reliabilities for each subscale were as follows: In Sample 1A, *α* = .82 (intrinsic), .79 (identified), .82 (positive introjected), .90 (negative introjected), .82 (external), and .87 (amotivation); in Sample 1B, *α* = .86 (intrinsic), .77 (identified), .83 (positive introjected), .92 (negative introjected), .84 (external), and .85 (amotivation). Figure 1 illustrates the average levels of each type of motivation.

**Table 1.** Final Items in the Autonomous Motivation for Romantic Relationships Scale.

Responses
Intrinsic
1. Because I enjoy being in a relationship.
2. Because I feel good being in a relationship.
3. Because being in a relationship is interesting.
4. Because being in a relationship is fun.
Identified
5. Because I strongly value being in a relationship.
6. Because being in a relationship is personally important to me.
7. Because it is my personal choice to be in a relationship.
8. Because being in a relationship is meaningful to me.
Positive introjected
9. Because I would feel proud of being in a relationship.
10. Because I want to prove to myself that I am capable of being in a relationship.
11. Because being in a relationship boosts my self-esteem.
12. Because I want to feel good about myself by being in a relationship.
Negative introjected
13. Because not being in a relationship would make me feel like a bit of a loser.
14. Because not being in a relationship would make me feel like there’s something wrong with me.
15. Because I would feel like a failure if I wasn’t in a relationship.
16. Because I don’t want to feel bad about myself by not being in a relationship.
External
17. Because it would make the people close to me happy if I were in a relationship.
18. Because it would be easier to maintain my social connections if I were in a relationship.
19. Because the people I value would like to see me in a relationship.
20. Because being in a relationship would make my relationships with my friends easier.
Amotivation
21. There is no reason I want to be in a relationship.
22. There is nothing that really motivates me to be in a relationship.
23. There may be good reasons to be in a relationship, but personally I’m not sure what they are.
24. I can’t think of anything that makes a relationship something I aspire to.

*Note*. Participants responded to the items after seeing the instructions, “to the extent that you would currently like to be in a romantic relationship, why is that?” and were provided the response options ranging from 1 (*strongly disagree*) to 5 (*strongly agree*).

### Measures: Individual Differences in Social Motivation

#### Attachment Style

Variation in *attachment avoidance* reflects individuals’ draw toward vulnerability and intimacy (e.g. Spielmann, Maxwell, et al., 2013), with higher levels of attachment avoidance characterized by less interest in romantic partnership in particular (Gillath et al., 2017; MacDonald & Park, 2022). Variation in *attachment anxiety* is related to the degree to which individuals experience insecurity about their worthiness of love (Mikulincer & Shaver, 2010). Individuals higher in attachment anxiety report particularly strong desire for romantic partnership (MacDonald & Park, 2022), which may be a pathway for quelling self-doubts and providing a tool for emotion regulation in general. Participants completed the 9-item Experiences in Close Relationships Revised scale to assess global attachment insecurity (Fraley et al., 2000). This scale consists of two subscales assessing attachment avoidance (e.g. “I don’t feel comfortable opening up to others”, αs > .79) and attachment anxiety (e.g. “I’m afraid that other people may abandon me”; αs > .86). This measure was completed on a 7-point response scale (1 = *strongly disagree* to 7 = *strongly agree)*.

#### Social Goals

Individuals vary in their goals for social relationships in general. *Approach social goals* reflect goals to create and capitalize on positive relationship outcomes, such as closeness and intimacy, and have been found to be associated with positive attitudes about relationships (Gable, 2006). *Avoidance social goals* reflect a focus on avoiding punishment and undesirable outcomes such as conflict, and have been found to be associated with less satisfaction with social relationships and negative attitudes toward relationships (Gable, 2006). Research has shown that individuals high in avoidance goals were equally high in life satisfaction whether single or partnered, whereas (in one of two studies) individuals high in approach goals were higher in life satisfaction when partnered than single (Girme et al., 2016). Participants completed the Social Goals Questionnaire (Gable, 2006). The scale consists of two 4-item subscales assessing approach (e.g. I try to deepen my relationships with my close others”; αs > .86) and avoidance (e.g. “I try to avoid disagreements and conflicts with people close to me; αs > .75) motives. This measure was completed on a 7-point response scale (1 = *strongly disagree* to 7 = *strongly agree)*.

#### Fear of Being Single

Positively related to attachment anxiety, *fear of being single* (FOBS) reflects concern, anxiety, or distress regarding one’s state of being without a romantic partner (FOBS; Spielmann, MacDonald, et al., 2013). Higher levels of fear of being single are related to less selective strategies for partner pursuit such as interest in partners lower in responsiveness (Spielmann, MacDonald, et al., 2013) and continued interest in ex-partners (Spielmann & Cantarella, 2020). Participants completed the 6-item Fear of Being Single scale (e.g. “I feel anxious when I think about being single forever; αs > .83; Spielmann, Maxwell, et al., 2013). Each item was rated on a scale ranging from 1 (*not true at all*) to 5 (*very true*).

#### Sociosexual Orientation

Sociosexual orientation captures variability in the extent to which individuals pursue sexual experiences in the context of casual, uncommitted sexual relationships versus more committed relationships (Simpson & Gangestad, 1991). Participants completed the revised Sociosexual Orientation Inventory (Penke & Asendorpf, 2008), which consists of nine items assessing general tendencies to accept, desire, or have casual sexual relationships (e.g., “With how many different partners have you had sex within the past 12 months”). Items were assessed on a 9-point scale with anchors varying depending on the item (αs > .82).

#### Communal strength (in Sample B only)

Communal strength reflects caretaking motivation or the desire to respond to the needs of and provide care for close others (Mills et al., 2004). Participants completed the 10-item Communal Strengths Scale (Mills et al., 2004) to assess the amount of motivation one has to respond to the needs of a communal partner (α = .70). Participants were given the instruction, “please answer the following questions based on how you would think, feel, and act if you had a romantic partner.” The question stems were modified to begin with the phrase “if you had a romantic partner.” (e.g. If you had a romantic partner, how happy would you feel when doing something that helps them?). This measure was completed on a 11-point response scale (0 = *not at all* to 10 = *extremely*).

### Measures: Romantic Desire and Interest

#### Commitment Readiness

*Commitment readiness* reflects one’s degree of orientation toward a committed romantic relationship (Agnew et al., 2019). Commitment readiness underlies one’s receptivity to relationship development and denotes a greater sense of preparedness for a relationship according to Relationship Receptivity Theory (Agnew et al., 2019). Hadden et al. (2018) explicitly show that commitment readiness is linked to a greater interest in developing a close romantic relationship and the active pursuit of initiating a relationship. Participants completed the eight-item commitment readiness measure (Agnew et al., 2019) to assess their preferences for a committed romantic relationship (e.g. “I feel ready to be involved in a committed relationship”; αs > .93). Each item was rated on a seven-point response scale (1 = *do not agree at all* to 6 = *completely agree)*.

#### Desire for a Partner

*Desire for a partner* reflects one’s current level of motivation for obtaining a romantic partner (MacDonald & Park, 2022). Research has demonstrated that people with higher desire for a partner report lower satisfaction with singlehood (Park et al., 2021) as well as less voluntary singlehood (Adamczyk, 2017; DePaulo, 2017; Kislev, 2020), and men with higher partner desire are more likely to subsequently enter a relationship (Sanderson et al., 2007). Participants completed a five-item measure to assess their level of desire or overall motivation to be in a romantic relationship (e.g. “My ideal right now is to be in a romantic relationship”; αs > .92; MacDonald & Park, 2022). Each item was rated on a seven-point response scale (1 = *strongly disagree* to 7 = *strongly agree)*.

#### Interest in Serious Relationships

Participants responded to the question, “How much are you interested in a serious romantic relationship?” on a scale ranging from 1 (*not interested)* to 7 (*very interested)*. This item was developed for use in the present study and has not been validated.

#### Interest in Casual Relationships

Participants responded to the question, “How much are you interested in a casual sexual or dating relationship?” on a scale ranging from 1 (*not interested*) to 7 (*very interested*). This item was developed for use in the present study and has not been validated.

### Analytic Strategy

All analyses were conducted in R (R Core Team, 2024). To test differences in the correlations across samples, we ran a series of models regressing each variable on each motivation type, including an interaction term between motivation and sample membership. Only 6 out of 60 interactions were significant, suggesting that the pattern of correlations was generally consistent across samples. Given the similarity in results, we ran our final analyses using a pooled sample, computing correlations between the motivation types and each of the measures, controlling for dummy coded variables for sample membership. The six correlations that appeared different across samples are noted in the table (also see Supplemental Tables S7 and S8 for separate results in each sample).

## Results

Descriptive statistics and correlations between the motivations are presented in Table 2. Table 3 displays the correlations between the motivations and related constructs. Given our large sample size (with which |*r*| as small as .10 reaches significance at *p* < .001), we focus on interpreting associations that were *r* = .10 or larger.

**Table 2.** Descriptive Statistics and Zero-Order Correlations Among the Subscales of Autonomous Motivation for Romantic Pursuit Scale (Study 1).

AMRPS motivation	*M* (*SD*)	1	2	3	4	5
1. Intrinsic	3.66 (0.79)					
2. Identified	3.34 (0.88)	.63				
3. Positive introjected	2.84 (1.05)	.38	.50			
4. Negative introjected	1.86 (1.00)	.12	.23	.60		
5. External	1.85 (0.88)	.04	.15	.41	.54	
6. Amotivation	2.51 (0.98)	-.53	-.52	-.31	-.16	-.02

*Notes. N* = 1,280. Response options ranged from 1 (*strongly disagree*) to 5 (*strongly agree*). All correlations are significant at *p* < .01 except for those between Intrinsic and External and External and Amotivation. *M* = mean; *SD* = standard deviation.

**Table 3.** Zero-Order Correlations Among Study Variables (Study 1).

AMRPS motivation	Att.Anx	Att.Avo	App.G	Avo.G	FOBS	SO	CS	Romantic desire/interest			
								Readiness	Desire	Serious	Casual
Intrinsic	.08**	−.22**	.29**	.07*	.17**	.06*	.23**	.47**	.54**	.55**	.14**
Identified	.10**	−.16**	.26**	.12**	.29**	−.07**	.25**	.48**	.59**	.58**	.04
Positive introjected	.22**	−.05	.04	.10**	.46**	.06*	.11**	.27**	.40**	.34**	.16**
Negative introjected	.25**	.04	−.08	.03	.52**	.03	.06	.10**	.24**	.18**	.13**
External	.14**	.04	−.09	.03	.33**	.01	.03	.02	.13**	.08**	.08**
Amotivation	−.06*	.16**	−.24**	−.07*	−.20**	.00	−.25**	−.51**	−.58**	−.53**	−.08**

*Notes. N* = 1,280. Correlations based on a pooled sample are presented (separate results in each sample can be found in the Supplemental Material Table S7 and S8). Six correlations that significantly differed across samples are underlined; correlations of avoidance goals with identified, positive introjected, and amotivation were significant in only one of the samples. Other three correlations only differed in the magnitude. Correlations with Communal Strength is based on Sample 1B only. Att.Anx = attachment anxiety; Att.Avo = attachment avoidance; App.G = approach social goals; Avo.G = avoidance social goals; FOBS = fear of being single; SO = sociosexual orientation; CS = communal strength; Readiness = commitment readiness; Desire = desire for a partner; Serious = interest in serious relationships; Casual = interest in casual relationships.

* *p* < .05. **p < .01.

### Attachment Orientations

Attachment anxiety was associated with higher levels of positive (*r* = .22) and negative introjected (*r* = .25), and external motivations (*r* = .14). These results portray anxiously attached individuals as desiring a relationship for relatively nonintrinsic reasons, consistent with the notion that they pursue relationship experiences as a means to an end, such as self-validation, rather than for the experiences themselves (e.g. Philippe et al., 2017). On the other hand, attachment avoidance was associated with lower levels of intrinsic (*r* = −.22) and identified motivation (*r* = −.16) as well as higher levels of amotivation (*r* = .16). These results are consistent with the notion that avoidant individuals value or prioritize intimacy less in their relationships (Ren et al., 2017) and as such may have less intrinsic motivation for romantic relationships.

### Social Goals

Endorsement of approach social goals was associated with higher levels of intrinsic (*r* = .29) and identified motivation (*r* = .26) as well as lower levels of amotivation (*r* = −.24). Avoidance social goals, on the other hand, showed weaker correlations with the motivations, with the only one stronger than > .10 being a positive relationship with identified motivation (*r* = .12). In addition, as indicated in Table 3, this was one of the correlations that did not replicate across samples.

### Fear of Being Single

Fear of being single showed significant relationships with all motivations, with particularly strong associations with less autonomous motivations such as introjected (*r* = .46 and .52 for positive and negative, respectively) and external motivations (*r* = .33). Overall, these relationships seem to suggest a degree of global draw toward relationships among those high in fear of being single with a particular appeal around possible resolution of self-worth and belonging concerns.

### Sociosexual Orientation

The six motivations showed weak relations with sociosexual orientation.

### Communal Strength

Communal strength correlated with higher levels of intrinsic (*r* = .23), identified (*r* = .25), and positive introjected motivation (*r* = .11) as well as lower amotivation (*r* = −.25). That is, those who report being motivated to care for romantic partners appear to be motivated for partnership for reasons more (but not exclusively) on the autonomous end of the measure.

### Romantic Desire and Interest

The six motivations were significantly related to the four indices of romantic desire/interest, but not so strongly as to suggest that they are redundant. In particular, general desire for a relationship, commitment readiness, and interest in a serious relationship were all related to higher levels of intrinsic (*r*s > .47), identified (*r*s > .48), and introjected motivations (*r*s > .27 and .10 for positive and negative) and lower levels of amotivation (*r*s > −.51). They were relatively weakly related to higher levels of external motivation (with general desire the only one with *r* > .10). Interestingly (and somewhat consistently with weak relations between the motivations and sociosexual orientation), interest in casual relationships showed weaker relationships with all the motivations (*r*s < .16).

## Discussion

Study 1’s results suggest that the AMRPS has potential utility as a tool to help organize the literature on motivation to pursue romantic relationships. Social motivations that are arguably tied to using relationships to shore up self-worth (attachment anxiety, fear of being single) appeared to have stronger connections with introjected and extrinsic motivations. That is, the data from AMRPS suggests that variables like fear of being single may represent the degree to which singles use relationship pursuit to attempt to manage feelings of pride/shame and please friends and family more so than to forge an intimate connection. On the other hand, approach social goals, a construct related to desires to deepen emotional intimacy, was particularly tied to higher intrinsic and identified motivation as well as lower amotivation. Relatedly, attachment avoidance, which has been shown to be related to experiencing less reward from intimate relationships (Gere et al., 2013), showed inverse relations to these three levels of motivation. Thus, the AMRPS seemed to be useful for identifying individual differences related to motivation to pursue romantic relationships because of the (lack of) reward of intimacy.

Study 1 also provided some evidence of discriminant validity. Avoidance social goals were not consistently related to either more or less autonomous motivation for relationship pursuit. This null finding is arguably consistent with Girme et al.’s (2016) finding that individuals higher in avoidance goals were equally happy partnered or single suggesting less differentiation between single and partnered status. Further, the various motivations captured by the AMRPS were consistently unrelated to sociosexual orientation. This suggests that the AMRPS reflects more the pursuit of romantic relationships than sexual experiences, reinforcing the inappropriateness of Gravel et al.’s (2016) Sexual Motivation Scale for our research goals. Indeed, given that the AMRPS was more strongly related to interest in serious rather than causal relationships, the data suggest that AMRPS chiefly reflects motivation for committed rather than short-term relationships.

The AMRPS was related to desire for a relationship relatively strongly at all levels of motivation, but especially at the intrinsic, identified, and amotivation levels. Indeed, although much of singlehood studies tends to focus on the social pressures that drive singles to partner (DePaulo, 2023), singles in our studies report intrinsic and identified motivations to be considerably stronger than extrinsic motivation. Relationship readiness was particularly tied to the more autonomous forms of motivation. It may be interesting for future research to examine if singles interpret feelings of relationship desire emanating from what SDT describes as the *true self* as a sign that they are ready for a relationship.

## Study 2

Overall, Study 1 suggests that a SDT framework provides a potentially useful means of organizing a variety of constructs related to motivation for pursuing romantic relationships. However, we believed that a particularly strong test of the framework’s utility lies in its potential to predict who is most likely to enter a romantic relationship. SDT theory and research suggests that individuals who are more autonomously motivated toward a goal are more persistent in pursuit of that goal and more likely to reach it successfully (Ryan & Deci, 2017, 2000). In Study 2, we examined the predictive validity of the AMRPS by tracking single individuals over time to examine the scale’s relation to stable singlehood/transitions into romantic relationships. We expected the new scale to be able to predict relationship transition, although we formed no specific a priori hypotheses regarding each subscale. However, given the possibility that any predictive power of higher levels of motivation may simply reflect general effects of higher overall well-being (a predictor of entering relationships; Braithwaite & Holt-Lunstad, 2017), we also examined if our motivation subscales can predict entering a relationship above and beyond life satisfaction. We further examined the predictive power of the AMRPS over and above other measures of romantic interest and intentions (desire for a partner, interest in a casual relationship, interest in a serious relationship, and intentions to start a relationship).

## Methods

### Participants and Procedure

A sample of 4,746 participants who were between the ages of 18 and 39 (premised on the notion that participants in this age range were more likely to start a new romantic relationship; Park et al., 2022) and were not currently in a relationship was recruited from Prolific in January 2022. The sample size was determined based on an a priori power analysis conducted to estimate a minimum sample size required to detect a small-sized effect if we were to run a regression model in a subset of participants who entered a relationship. Considering varying attrition rates (~25%) and the proportion of individuals entering a relationship (~30%), we decided to recruit at least 4,000 individuals. Our attrition rate was informed by previous research conducted on the same recruitment platform (e.g. Kothe & Ling, 2019), and transition rate was based on previous studies following single individuals for 5 months (e.g. Gerlach et al., 2019; 34%).

After excluding participants who did not complete the study, failed attention checks, or reported being dishonest in answering the questions, the final sample consisted of 4,020 participants. The present analyses are based on a subset of individuals (*n* = 3,186; 79% retention rate) who also participated in our follow-up survey. Specifically, 6 months after the baseline survey (including periodic reminders to participants the follow-up survey was upcoming), participants were re-contacted via the Prolific messaging system and notified that the follow-up survey was available to complete over a two-week period. Our analytic sample comprised 1,583 males, 1,590 females, and 13 individuals with other or unidentified (i.e. nonreported) gender identities. Participants were 24.62 years old on average (*SD* = 5.08). Participants described their ethnic/racial background (multiple responses allowed) as White (n = 1,945), Latino/Hispanic (*n* = 472), African (*n* = 417) East Asian (*n* = 157), South Asian (*n* = 124), Middle Eastern (*n* = 101), Caribbean (*n* = 27), or other (*n* = 145). Participants identified as heterosexual (n = 2,359), with others identifying as bisexual (*n* = 420), uncertain or questioning (*n* = 166), gay (*n* = 135), queer (*n* = 100), lesbian (*n* = 80), or other/refuse to answer (*n* = 130). Most participants had previously been in a romantic relationship (n = 2,237), which ended an average of 29.29 months before the start of the study (*SD* = 29.34; range = 3–336). Participants responded to a battery of questionnaires that included the AMRPS and measures listed below. The reliabilities for the AMRPS were as follows: α = .83 (intrinsic), .78 (identified), .83 (positive introjected), .93 (negative introjected, .85 (external), and .88 (amotivation). Questionnaires were all presented in English, and the survey was available to Prolific users regardless of geographical location.

### Baseline Measures

#### Desire for a Partner

Participants responded to a single item, “I want to have a romantic partner,” on a 7-point scale (1 = *strongly disagree* to 7 = *strongly agree*).

#### Interest in Serious Relationships

The same single-item measure was used as in Study 1.

#### Interest in Casual Relationships

The same single-item measure was used as in Study 1.

#### Intentions to Begin a Romantic Relationship

Participants responded to the question, “To what extent do you intend to begin a romantic relationship in the next six months?” on a scale ranging from 1 *(not at all*) to 8 (*a lot*).

#### Life Satisfaction

Participants responded to the question, “In general, how satisfied are you with your life?” on a four-point scale ranging from 1 (*very dissatisfied*) to 4 (*very satisfied*).

### Outcome: Relationship Status

At the follow-up, participants were asked, “Are you currently in a romantic relationship” to which they could answer “*yes*” or “*no*.” If participants selected “*no*,” they were asked, “In the past 6 months, have you been in a romantic relationship?” to which they could answer “*yes*” or “*no*.” About 25% of the sample (*n* = 798) had experienced at least one relationship transition. Note that some of these participants had subsequently exited that relationship, leaving about 15% of the sample (*n* = 472) reporting currently being in a relationship. The pattern of results across these two outcomes were equivalent: We conducted *z*-tests to compare the odds ratios for each motivation across the two models (i.e. one treating individuals whose relationships ended as partnered and the other treating them as single). None of the differences were statistically significant (|*z*| < 1.05). Although we report the results in terms of those who entered a relationship at all since the baseline, we present results from the set of models predicting the participant being in a relationship at Time 2 in the Supplemental Materials (Table S10).

### Outcome: Relationship Quality

For exploratory purposes, we asked participants who were currently in a relationship at Time 2 about their satisfaction with the current relationship (“I feel satisfied with our relationship”), investment (“I have put a great deal into my relationship that I would lose if the relationship were to end), quality of alternatives (“My alternatives to this relationship are very attractive to me [dating another, being single, etc.]”), and commitment (“I am committed to maintaining my relationship with my partner”). These single-item measures capture the four components of Rusbult’s investment model (Rusbult et al., 1998) and were selected from the full scale to lessen participant burden. All items were rated on a 9-point scale, ranging from 0 (*do not agree at all*) to 8 (*agree completely*).

## Analyses and Results

### Primary Analyses

Because our primary outcome measure was a dichotomous relationship status variable, we conducted a series of logistic regression models predicting relationship status at Time 2 with different sets of predictors. The first model included the six motivations from the AMRPS as predictors, the second model additionally included life satisfaction, and the third model included other measures of romantic desire and intentions. All models included gender and age as covariates. We interpreted an Odds Ratio (OR) higher than 1 as indicating that higher values of the predictor variable were associated with higher odds of having entered a relationship.

Table 4 shows that in the first model, intrinsic (*OR* = 1.47) and identified motivation (*OR* = 1.35) emerged as significant predictors of relationship status such that those who had stronger intrinsic or identified motivation for dating were more likely to have entered a romantic relationship. Those who endorsed negative introjected motivation more strongly at baseline were less likely to have entered a relationship (*OR* = 0.85). These effects were attenuated but remained significant in Model 2 when we controlled for general life satisfaction and in Model 3 controlling for other measures of romantic desires and intentions. Surprisingly, in Model 3, those who endorsed amotivation more strongly at baseline appeared more likely to have entered a relationship (*OR* = 1.17). Consistent with the idea that intention is an immediate antecedent of a behavior (Ajzen, 2020), people’s reported intentions for starting a relationship at baseline appeared to be a strong predictor of relationship status at Time 2 (*OR* = 1.38).

**Table 4.** Results from the Logistic Regression Predicting Having Entered a Relationship by Time 2.

Predictors	Model 1			Model 2			Model 3		
	*OR*	*z*	*p*	*OR*	*z*	*p*	*OR*	*z*	*p*
Sex (female)	1.13	1.46	.15	1.13	1.39	.16	1.12	1.25	.21
Sex (other)	0.92	−0.13	.90	0.92	−0.12	.91	1.07	0.10	.92
Age	0.98	−2.20	.03	0.98	−2.12	.03	0.98	−1.93	.05
AMRPS									
Intrinsic	1.47	4.63	<.001	1.46	4.57	<.001	1.32	3.18	.001
Identified	1.35	4.27	<.001	1.35	4.27	<.001	1.23	2.67	.008
Positive introjected	0.90	−1.73	.08	0.90	−1.71	.09	0.89	−1.85	.06
Negative introjected	0.85	−3.12	.002	0.86	−2.87	.004	0.85	−2.92	.004
External	0.99	−0.17	.82	0.99	−0.22	.83	0.95	−0.98	.33
Amotivation	1.09	1.60	.11	1.09	1.64	.10	1.17	2.69	.007
Well-being									
Life satisfaction				1.05	1.11	.27			
Romantic desire									
Desire for partner							0.98	−0.33	.74
Casual interest							1.00	−0.11	.91
Serious interest							0.89	−2.59	.01
Intentions							1.38	10.76	<.001

*Notes. N* = 3,186. An Odds Ratio (OR) higher than 1 indicates that higher values of the predictor variable were associated with higher odds of having entered a relationship.

### Additional Analyses

#### Variable Importance

**Figure 2.**
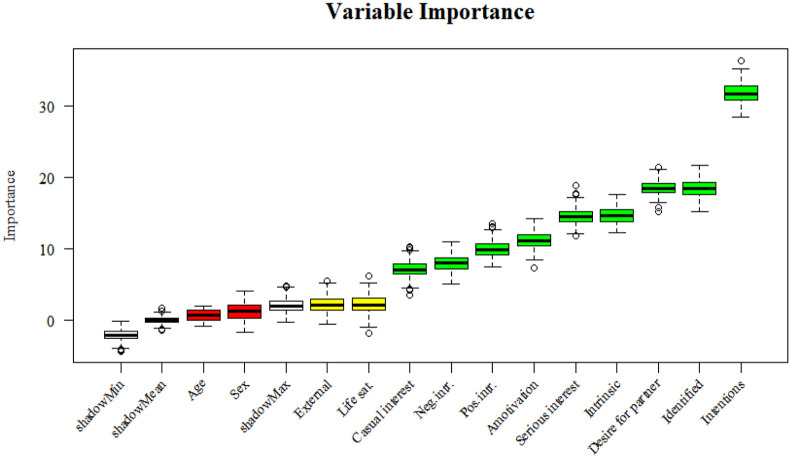
Importance of predictor variables in model 3 based on the Boruta algorithm. *Notes*. White boxplots represent the importance of shadow features (see Kursa & Rudnicki, 2010). Variables with importance statistically higher (lower) than the shadow variables are considered important and are in green (red). Variables that could not be classified as either important or unimportant after 500 iterations are colored yellow.

To gain additional insights into the importance of each variable in predicting relationship transitions, we used the Boruta algorithm (the *Boruta* package; Kursa & Rudnicki, 2010), a feature selection method based on the random forest classifier. Essentially, the Boruta algorithm runs multiple random forests to provide robust estimates of variable importance (i.e. how much the model’s accuracy decreases when the values of a given variable are randomly shuffled). Additionally, it compares the importance of the real predictors to that of random “shadow attributes,” allowing us to statistically determine whether each variable makes a meaningful contribution. Figure 2 suggests that nine of our 16 predictors in Model 3 were considered as “important” predictors of relationship transitions (in green); among the AMRPS motivations, the most important was identified motivation, followed by intrinsic motivation, amotivation, and positive and negative introjected motivations.

#### Predicting Relationship Quality Among Partnered Individuals

We examined if the motivations at baseline can predict the quality of the relationship people entered by running four sets of models (similar to Model 1 in Table 4). The only significant association was that between amotivation and quality of alternatives (*b* = 0.47, *SE* = 0.14, *p* < .001), suggesting that singles who more strongly endorsed amotivation perceived higher quality of alternatives to their relationship. Full results are available in the Supplemental Materials (Table S11).

## Discussion

The results of Study 2 support the notion that higher levels of autonomous motivation are linked with greater likelihood of partnering. Individuals who reported higher levels of intrinsic or identified motivation were more likely to transition from singlehood to partnered status after 6 months, even accounting for a wide range of plausible covariates. Thus, consistent with past SDT research in multiple domains (e.g. Koestner et al, 2008), individuals who reported more autonomous motivation for romantic partnering appeared to have more successfully accomplished their goal. Further, individuals with higher negative introjected motivation, which was significantly related to higher fear of being single and anxious attachment, were significantly less likely to enter a relationship. That is, those who reported being motivated to pursue romantic relationships to enhance their own self-image appear to be relatively less likely to partner. On the one hand, this may be surprising given the strong desire for a relationship on the part of those with anxious attachment and fear of being single, and the strong effort and lower standards they appear to bring to dating (Brumbaugh & Fraley, 2010; Spielmann, MacDonald, et al., 2013). However, these insecurities are also accompanied by emotional reactivity and sensitivity to rejection that have been shown to interfere with success in attracting partners (McClure et al., 2014).

Unexpectedly, singles who scored higher on the amotivation scale were also somewhat more likely to change to partnered status in one of our more stringent regression analyses. Of note, the analyses reported in that particular test only examined the effects of each of the motivations when entered simultaneously. When amotivation was tested as a predictor of relationship status alone (i.e. not controlling for the other motivations), higher levels of amotivation predicted lower odds of having entered a relationship (see Supplemental Materials Table S9). This suggests a unique aspect of amotivation whereby one part of its variance contributes to less likelihood of entering a relationship, but another part of its variance contributes to more likelihood of entering a relationship. Although we can only speculate, one possibility is that one contributor to reporting amotivation for a romantic relationship (besides low interest in sexual/romantic connection) may be that one is romantically/sexually desirous but is meeting those needs outside of committed relationships (e.g., through uncommitted sexual encounters). Indeed, amotivated individuals who transitioned to partnered status reported relatively high quality of alternatives to that relationship. Further, the amotivation scale was significantly, positively correlated with singles’ sexual satisfaction (see Supplemental Materials), suggesting that a number of amotivated participants may be sexually active. In fact, Park et al. (2021) showed that although sexually satisfied singles reported lower levels of desire for a relationship they were also more likely to transition into a relationship over time than less sexually satisfied singles. The tendency for sexually active singles with low reported desire for committed relationships to transition into such relationships over time may be an example of what Joel and MacDonald (2021) have called a progression bias, or the tendency for romantic entanglements to progress from lower to higher levels of commitment. Thus, to the extent that one goal of the current research was to be able to highlight and understand amotivation for pursuit of romantic relationships, future research may need to separate out sexually active amotivated individuals from those who are not sexually active.

Exploratory analyses indicated that the various levels of motivation were almost completely unrelated to reported quality of these relationships. Given that all relationships were 6 months in length or less, future research following individuals past the “honeymoon stage” (e.g. Joel & Machia, 2024) would be of interest. Overall, Study 2 appears to support the validity of the AMRPS by demonstrating via longitudinal data that individuals who report more autonomous motivation for romantic partnering are, indeed, more likely to become part of a committed partnership (notably, these results also similarly support the validity of the measures of interest in a serious relationship and intentions to begin a relationship).

## General Discussion

The results of our two studies suggest that SDT may be a useful perspective for consolidating a variety of potential motivations (or lack thereof) for pursuing a romantic relationship into one theoretically derived framework that captures variation in autonomous motivation. Study 1 provided evidence of the utility of the SDT framework by demonstrating the scale’s relations to various measures of relationship desires and motivations, including highlighting that the motivation captured by the AMRPS appears to be primarily desire for committed (rather than casual) relationships. Study 2 demonstrated that the AMRPS is useful for predicting who is likely to enter a romantic relationship. That is, consistent with the SDT notion that more autonomous motivation is associated with more persistence and success in goal pursuit, singles higher in intrinsic and identified motivation, as well as lower in negative introjected motivation, were more likely to report having transitioned to a romantic relationship. Curiously, there was also a significant finding when examining unique statistical effects of each motivation such that singles higher in amotivation were also more likely to enter a relationship. We speculate this might mean that amotivation can reflect both low relationship/sexual desire as well as relationship/sexual desire that is being met outside of committed relationships.

One goal of developing the AMRPS was to provide a unifying theoretical framework for organizing the growing literature on motivation for pursuing romantic relationships. The scale appears to be useful for this purpose in a way that can add to our theoretical understanding of such relational pursuit constructs. For example, individuals who report high commitment readiness appear to hold largely autonomous motivation for relationships, suggesting that commitment readiness may primarily reflect anticipated enjoyment of relationships and the achievement of personally valued goals. On the other hand, fear of being single (FOBS) related strongly to introjected and external motivation, suggesting that FOBS may reflect seeing relationships as a tool for shoring up self-worth and cementing relationships with friends and family. Future research may benefit from expanding the list of constructs examined in light of the AMRPS to include endorsement of variables such as relationship ideology (e.g. Day et al., 2011), perceptions of social stigma against singlehood (Girme et al., 2022), or desire for children (Rholes et al., 2006).

One important research goal highlighted by the emergence of singlehood studies is to identify when people are more or less likely to remain single versus transition into a relationship. The current data shed light on this question by recognizing that the motivations that are more likely to be associated with a transition into a romantic relationship are those where romantic relationships are valued for the rewards they bring and those that support relationships as a personally valued life goal. On the other hand, motivations to pursue relationships tied to perceptions of external pressure were not related to likelihood of partnering in our data. To date, singlehood scholarship has largely focused on the personal costs that can come from social pressure to partner (Day et al., 2011; DePaulo & Morris, 2005; Sprecher & Felmlee, 2021), such as the feelings of social stigma that have the potential to undermine singles’ well-being (Girme et al., 2022). However, the extent to which such pressure actually succeeds in moving people from singlehood to couple relationships has been an open question. Our data suggest the possibility that, at least when such pressure to partner is only partially internalized, it may not be particularly likely to lead individuals to actually transition into a romantic partnership (at least in the largely Western context in which we collected our data). Instead, conditions that lead individuals to find personal, intrinsic value in romantic relationships may be more likely to lead to transitions from singlehood to partnering.

Given the importance of autonomous motivation for romantic partnering that is suggested by our research, what might be the origin of such motivation? According to SDT, autonomous motivation facilitates and is facilitated by basic psychological need satisfaction (Reis et al., 2000; Sheldon et al., 1996). The three needs posited by SDT are autonomy, competence, and relatedness (Ryan & Deci, 2017, 2000). The need for autonomy refers to one feeling as though one is the originator of one’s behavior, the need for competence refers to one feeling effective and capable of achieving the desired outcome, and the need for relatedness refers to feeling connected to others. According to SDT, individuals are more likely to behave in ways consistent with their true self once these needs are met, as reflected in more autonomous motivation for goal pursuit. That is, contextual and relational support for these three needs is what facilitates internalization and more autonomous forms of self-regulation (Deci et al., 1994; Pelletier & Rocchi, 2023), including for romantic goals (e.g. Knee et al., 2013; Rodriguez et al., 2015). As such, singles for whom motivation to partner is congruent with what SDT calls their true self should feel more of a sense of choice in their dating pursuits. Furthermore, those who feel more skilled at relationship pursuit and who feel more social support for their idiosyncratic dating goals would appear to be more likely to develop the more autonomous motivation for relationship pursuit that our results suggest predicts greater likelihood of transition out of singlehood. Indeed, the stronger endorsement of intrinsic and identified motivations relative to extrinsic motivation suggests, from a SDT perspective, that motivation for partnership may be an important part of many singles’ true self. What variables promote feelings of autonomy, competence, and relatedness in the context of relationship pursuit appears to be a fruitful avenue for future research (Girme et al., 2023).

These considerations suggest that motives for pursuing relationships may not be static, but may well change over time as one’s development and social environment changes. For example, in their update of Maslow’s (1943) hierarchy of needs model, Kenrick et al. (2010) argue for a hierarchy wherein the motivation to acquire a mate emerges after motives for affiliation and status/esteem, in that order, are satisfied. This suggests that individuals who are at the stage of attempting to satisfy affiliation needs may be more likely to pursue romantic relationships for more extrinsic reasons (e.g. family and friend approval). When affiliation needs are satisfied and status/esteem motives emerge more strongly, individuals may be more likely to pursue romantic relationships for more introjected reasons (e.g. to feel valuable). Finally, when status/esteem motives are satisfied and mate acquisition becomes a stronger goal in and of itself, motives for relationship pursuit may become more identified and/or intrinsic. This analysis is a reminder that when single individuals report dissatisfaction with singlehood, this may reflect a sense of deficit from the lack of a partner per se, but may also (or instead) reflect frustration with satisfying the conditions (i.e. social connection, feelings of self-worth) that Kenrick et al. ’s (2010) model suggests precede the emergence of intrinsic relationship desire. For example, although incels’ “presenting complaint” is the lack of a girlfriend, their social isolation and low self-esteem may be stronger contributors to their relatively low well-being (Sparks et al., 2022). In any event, future research examining how motives for partnering change alongside changes in development and social conditions would be highly valuable.

Relatedly, we believe that the AMRPS may be valuable for better understanding the process of seeking out potential partners. One important and consistent finding from SDT research is that higher levels of autonomous motivation for a goal are related to more persistence in pursuit of that goal (Koestner et al., 2008). As such we would expect singles who are more autonomously motivated for relationship pursuit to be less likely to experience, for example, dating app burnout (Sharabi et al., 2024). This does not mean that we would expect more autonomously motivated daters to be less selective however, as pursuit of the goal of a committed relationship would mean persistence in finding a partner suitable for achieving relationship satisfaction and stability. Indeed, fear of being single, which has been shown to be related to less selective partner choice (Spielmann, MacDonald, et al., 2013), is related to relatively lower levels of autonomous motivation for dating in our data.

In general, this line of thinking leads to questions as to whether different levels of motivation might be related to attraction to different characteristics in potential partners. For example, if intrinsic motivation is tied to finding relationships enjoyable, then characteristics that promote more hedonistic qualities of relationships (e.g. sense of humor) might stand out to intrinsically motivated daters. More identified daters, for whom relationships are a means to an important goal, might seek partners best suited to that particular goal (e.g. someone interested in having children). Having said that, the relationships of those who seek relationships based on identified motivation may be more susceptible to dissolution when that goal is threatened (e.g. a partner turns out to be unable to have children) even when the relationship itself is enjoyable. Introjected daters, for whom relationships are a means for shoring up self-worth, may find themselves particularly attracted to superficial markers of status such as attractiveness or popularity that they imagine will boost their own status. Extrinsically motivated daters may find themselves attracted to qualities that are valued by family and friends. Future research examining the relation between the AMRPS and the qualities sought in romantic partners thus seems potentially valuable.

It is also an open question to what extent individuals’ pre-relationship motivations for relationship pursuit are related to the quality of the relationships individuals eventually start. On the one hand, we did not find any relation between autonomous motivation for relationship pursuit and levels of satisfaction and commitment in relationships that were started. However, these earliest stages of a relationship are unique in that they are marked by strong infatuation (Joel & Machia, 2024), thus creating a strong situation that may initially mask underlying vulnerabilities. Indeed, research with more established relationships has suggested that higher levels of need satisfaction and autonomous motivation are associated with better relationship functioning (Blais et al., 1990; Knee et al., 2005). Our research raises the interesting question of how much of the positive role of autonomous motivation in established relationship functioning is related to pre-relationship dating motivation, and thus is a characteristic of the conditions that led to the relationship starting rather than the relationship per se.

### Strengths and Limitations

We believe this research is uniquely valuable in its theoretically based approach to understanding motivations toward romantic partnering in a fashion that recognizes such motivation can take a variety of forms and does not assume that romantic partnering is something that everyone desires. Our studies are well powered and our approach of examining variables that predict romantic partnering using longitudinal methods (with a nearly 80% retention rate) outside of a speed dating context is particularly rare in the relationship literature. As such, we believe these studies provide valuable insight both theoretically and methodologically.

However, our studies contain important limitations through which our results must be understood. First, our data primarily originate from Western countries (as well as English speakers in other countries), and thus, it is unclear how our results may generalize to other world regions in which relationship dynamics differ. For example, in cultural contexts marked by more traditional gender roles, different norms for relational intimacy may affect individuals’ expectations of the opportunities for relational enjoyment (Marshall, 2008), leading to different evaluations of intrinsic motivation. Further, in contexts where the decision to pursue relationships is more shared with family (e.g., MacDonald et al., 2012), less autonomous forms of motivation, such as external motivation, may play more of a role in predicting transition into a committed relationship. Research examining the dynamics of autonomous motivation for partnerhood across cultures is a crucial future direction.

One issue particular to Study 2 is that we specifically selected an age range of 18–39 on the premise that such individuals would be more likely to be seeking romantic partners than older individuals. Thus, we cannot be certain the extent to which the results of Study 2 would generalize to older populations. For example, research suggests that older men may particularly seek romantic partners not only for companionship but also for assistance with practical needs such as housework and medical care (Harris, 2023). As such, older men may very well be seeking opportunities to partner for reasons that are not fully autonomous. On the other hand, older women—who often report lifetimes of caring for others—are particularly averse to such caretaking and report seeking partnership only when doing so is personally gratifying (Harris, 2023). In this sense, the importance of autonomous motivation may in some ways become that much more foregrounded (and gendered) in older age.

Relatedly, it is important to note that although we strived to create scales at each level of motivation that broadly represented that construct, it was impossible to include all possible reasons for pursuing relationships, and as such, we do not claim that our scales represent the full breadth of reasons for relationship pursuit. Indeed, future research that attempts to map out a more complete range of different reasons individuals may seek romantic relationships would be of value and would be likely to highlight reasons that are not captured by the AMRPS (e.g. practical support such as medical care).

Another important point is that our data cannot speak to why various motivations are related to more or less likelihood of partnering. We find that autonomous motivation is more likely to be associated with the successful pursuit of a partner and it seems likely to us that there is an important contribution to this effect made by the oft-found goal pursuit effectiveness associated with autonomous motivation (e.g. autonomous daters may be more likely to persist even after dating failures). However, a unique aspect of partnering as a goal is that the object of the goal is themselves a person with choice and volition. As such, there may be characteristics associated with higher levels of autonomous motivation that make that person more attractive to others (e.g. physical attractiveness), which also help account for higher rates of partnering. Similarly, there may be aspects associated with less autonomous motivation (e.g. poor health) that are related to lower mate value, aside from that individual’s psychological motivations. Overall, then, our results should be taken as largely descriptive with future research needed to better support speculations of causality. Indeed, future research examining what exactly autonomously motivated daters do (or are) that contributes to their higher likelihood of partnering appears to be an important next step.

## Conclusion

People can have many different motivations to pursue romantic relationships, if they have any such motivation at all. We believe we have provided a framework and a useful measurement tool for accounting for this range of motivation. It is our hope that this tool proves valuable both for keeping relationship researchers mindful of the variety of starting places from which people approach the decision to partner with someone, as well as for documenting the associations these motivations have with the social lives and well-being of individuals.

## Supplemental Material

sj-docx-1-psp-10.1177_01461672251331699 – Supplemental material for Why Do You Want a Romantic Relationship? Individual Differences in Motives for Romantic Relationship Pursuit Supplemental material, sj-docx-1-psp-10.1177_01461672251331699 for Why Do You Want a Romantic Relationship? Individual Differences in Motives for Romantic Relationship Pursuit by Geoff MacDonald, Serena Thapar, William S. Ryan, Joanne M. Chung, Elaine Hoan and Yoobin Park in Personality and Social Psychology Bulletin

## Notes

1. The final form of external regulation that has become entirely internalized is *integrated* regulation. Here not only does one engage in the behavior because one finds it personally valuable and meaningful but also integrated within one’s sense of self and is part of one’s self-concept. Because, especially in some contexts, there is minimal distinction between identification and integration, researchers do not always explicitly include integration in their measurement of the motivational continuum (Sheldon et al., 2017).
