# Supplementary material for: Why Do You Want a Romantic Relationship? Individual Differences in Motives for Romantic Relationship Pursuit
Source: Pers Soc Psychol Bull. 2025 May 12;52(8):2230–46. doi: 10.1177/01461672251331699 (PMC13310306; doi:10.1177/01461672251331699)
Supplement: sj-docx-1-psp-10.1177_01461672251331699 – Supplemental material for Why Do You Want a Romantic Relationship? Individual Differences in Motives for Romantic Relationship Pursuit [file sj-docx-1-psp-10.1177_01461672251331699.docx]

**Supplemental Materials for** *Why Do You Want a Romantic Relationship? Individual Differences in Motives for Romantic Relationship Pursuit*

**Table of Contents**

[**Table S1** *Items in the Pilot Study (N = 500)* 3](#_Toc172725990)

[**Table S2** *Goodness-of-Fit Statistics and Information Criteria for Models Describing the Autonomous Motivations for Romantic Pursuit (Study S1)* 8](#_Toc172725991)

[**Table S3** *Standardized Factor Loadings (λ) and Uniquenesses (δ) for Confirmatory Factor Analysis and Exploratory Structural Equation Modeling for the Autonomous Motivation for Romantic Pursuit Scale (Sample S1A)* 9](#_Toc172725992)

[**Table** **S4** *Standardized Factor Loadings (λ) and Uniquenesses (δ) for Confirmatory Factor Analysis and Exploratory Structural Equation Modeling for the Autonomous Motivation for Romantic Pursuit Scale (Sample S1B)* 10](#_Toc172725993)

[**Table** **S5** *Standardized Factor Loadings (λ) and Uniquenesses (δ) for Bifactor Exploratory Structural Equation Modeling (B-ESEM) for the Autonomous Motivation for Romantic Pursuit Scale (Sample S1A)* 11](#_Toc172725994)

[**Table** **S6** *Standardized Factor Loadings (λ) and Uniquenesses (δ) for Bifactor Exploratory Structural Equation Modeling (B-ESEM) for the Autonomous Motivation for Romantic Pursuit Scale (Sample S1B)* 13](#_Toc172725995)

[**Table** **S7** *Correlations in Sample 1A (Study 1 in the Manuscript)* 15](#_Toc172725996)

[**Table** **S8** *Correlations in Sample 1B (Study 1 in the Manuscript)* 16](#_Toc172725997)

[**Table** **S9** *Results from the Logistic Regression Models Predicting Having Entered a Relationship by Time 2 (Study 2)* 17](#_Toc172725998)

[**Table** **S10** *Results from the Logistic Regression Models Predicting Being in a Relationship at Time 2 (Study 2)* 18](#_Toc172725999)

[**Table** **S11** *Results from the Regression Models Predicting Relationship Quality (Study 2)* 19](#_Toc172726000)

**Pilot Study (Scale Development) and Study 1 (Structural Validity)**

We drafted a 24-item scale of motivation for relationship pursuit based on previous SDT scale development research (Sheldon et al., 2017). Two of the current authors with expertise in both singlehood and romantic relationship research adapted each of the items (or developed a related item in cases where a direct adaptation was not possible) from the Sheldon et al. (2017) 24-item scale to suit a relationship pursuit context. Participants answered each item responding to the prompt, “To the extent that you would currently like to be in a romantic relationship, why is that?” with a scale ranging from 1 (*strongly disagree*) to 5 (*strongly agree*). Items that had low item to total correlations or little variability were revised following a pilot study testing these items. Table S1 shows the full list of items used in the pilot study.

# Table S1 *Items in the Pilot Study (N = 500)*

| ***Intrinsic*** |
| --- |
| 1. Because I enjoy being in a relationship. |
| 1. Because I feel good being in a relationship. |
| 1. Because being in a relationship is interesting. |
| 1. Because being in a relationship is fun. |
| ***Identified*** |
| 1. Because I strongly value being in a relationship. |
| 1. Because being in a relationship is personally important to me. |
| 1. Because it is my personal choice to be in a relationship. |
| 1. Because being in a relationship is meaningful to me. |
| ***Positive introjected*** |
| 1. Because I would feel proud of being in a relationship. |
| 1. Because I want to prove to myself that I am capable of being in a relationship. |
| 1. Because being in a relationship boosts my self-esteem. |
| 1. Because I want to feel good about myself by being in a relationship. |
| ***Negative introjected*** |
| 1. Because I would feel guilty if I wasn’t in a relationship. |
| 1. Because I would feel ashamed if I wasn’t in a relationship |
| 1. Because I would feel like a failure if I wasn't in a relationship. |
| 1. Because I don't want to feel bad about myself by not being in a relationship. |
| ***External*** |
| 1. Because it would make the people close to me happy if I were in a relationship. |
| 1. Because if I am not in a relationship others will get upset. |
| 1. Because people who are important to me will like me better if I am in a relationship |
| 1. Because I don’t have any choice but to get in a relationship |
| ***Amotivation*** |
| 1. There is no reason I want to be in a relationship. |
| 1. There is nothing that really motivates me to be in a relationship. |
| 1. There may be good reasons to be in a relationship, but personally I’m not sure what they are. |
| 1. I can’t think of anything that makes a relationship something I aspire to. |

*Notes.* Items in red showed little variability or low correlations with other items and were revised in the new scale. For external regulation, the revised scale focuses specifically on socially relevant motivations.

**Study S1**

In Study 1, we examined the underlying autonomy continuum using the bifactor exploratory structural equation modeling (ESEM) in two samples of single individuals. In contrast to a more commonly used confirmatory factor analysis (independent cluster model; CFA) which has a restrictive assumption that each item loads only on to a pre-determined factor, ESEM allows for minimal degrees of cross-loadings and is considered to align better with the multidimensional conceptualization of motivations (see Howard et al., 2018 for details about each model assumptions). In bifactor ESEM, the global factor (G-factor) captures the overall level of self-determined motivation while the specific factor (S-factor) captures the different types of the motivation that are left unexplained by the G-factor. Following Morin and colleagues’ (2016) recommendations, we contrast the results produced from specifying the bifactor ESEM to simpler alternative models: CFA, ESEM, and bifactor CFA. We describe each model specifications in the Data Analysis section below.

**Methods**

**Participants**

**Sample S1A.** A total of 707 participants who were at least 18 years old and were not currently involved in a romantic relationship were recruited from Prolific Academic. We excluded 24 participants who failed attention checks or who reported having been dishonest in answering the questions. The final sample consisted of 683 participants (454 men, 209 women, 8 transgender, 12 other or prefer not to disclose) who were 25.24 years old on average (*SD* = 8.35; range = 18 to 67). The majority of the sample identified their ethnic/racial background as Caucasian (*n* = 424), Latino/Hispanic (*n* = 143), East Asian (*n* = 35), Middle Eastern (*n* = 26), African (*n* = 21), South Asian (*n* = 23), and Caribbean (*n* = 3), or Other (*n* = 37).

**Sample S1B.** A total of 783 participants who were at least 20 years old and were not currently involved in a romantic relationship were recruited from Prolific Academic and completed our survey. We excluded 37 participants who failed attention checks or who reported having been dishonest in answering the questions. The final sample consisted of 746 participants (375 men, 371 women) who were 38.85 years old on average (*SD* = 11.65; range = 20 to 59). We did not ask about participant ethnicity. For both samples, the target sample size was determined based on a power analysis for other questions being examined in the data, but we ensured that the sample was large enough for exploring the bifactor structure of the model (based on a table approach; Hancock & French 2013, see Morin et al., 2020).

**Data Analysis**

All analyses were conducted using Mplus 8.5 (Muthén & Muthén, 1998-2017), using syntax adapted from Howard and colleagues (2018). Models were estimated using robust Maximum Likelihood estimator (MLR) and specified as follows: 1) CFA: items were only allowed to load on their respective type of motivation, with cross-loadings constrained to be zero; 2) ESEM: items were freely estimated to load on their respective type of motivation, with all cross-loadings also freely estimated but targeted to be as close to zero as possible; 3) bifactor CFA: all items were specified to load on the self-determination global factor as well as their respective type of motivation. No cross-loading was allowed; and 4) bifactor ESEM: item loadings were freely estimated on the global factor and on their respective motivation types. As for ESEM, cross-loadings were freely estimated but targeted to be as close to zero as possible.

Following previous ESEM applications (Howard et al., 2016; Marsh et al., 2014), model fit was evaluated using several goodness-of-fit indices and information criteria. Specifically, values greater than 0.90 for the comparative fit index (CFI) and Tucker-Lewis index (TLI) and values smaller than 0.08 for the root mean square error of approximation (RMSEA) were considered to indicate adequate fit; the Akaike information criterion (AIC), the constant AIC (CAIC), the Bayesian information criterion (BIC), and the sample-size adjusted BIC (ABIC) were used to compare alternative models, with lower values suggesting a better fit. In interpreting and comparing each model, we also closely inspected the standardized factor loadings, which were considered to be poor if smaller than .32 and very good if larger than .63 (Tabachnick & Fidell, 2001). It should be emphasized that cutoffs for evaluating the model fit or factor loadings are arbitrary, and the theoretical meaningfulness of the parameters in each model need to be examined when making comparisons.

**Results**

**Model evaluation**

Table S2 presents the goodness-of-fit indices and information criteria of the four models in Samples S1A and B. In both samples, ESEM solution seemed to provide a better representation than CFA according to goodness-of-fit indices (i.e., higher values). As shown in Table S3, overall, the size of the factor loadings of the items on their target factors were similar but lower in the ESEM solutions than in the CFA. There were some significant cross-loadings (but none larger than each item’s target loadings) in the ESEM solution, thus the factor correlations were reduced in the ESEM solution compared to the CFA solution.

Perhaps not surprisingly, when comparing the ESEM to its bifactor counterpart, the latter provided a better fit to the data in both samples. Particularly, in Sample S1B, confidence intervals for the RMSEA did not overlap between ESEM and B-ESEM, providing better support for the superiority of the latter model. Inspecting the model parameters in the B-ESEM solution also suggested that the structure fits theoretical expectations. Specifically, when looking at the model parameters in the B-ESEM solution (Tables 3 and 4), all indicators loaded significantly onto the global factor, and except for two items in Sample S1A, onto their respective specific factor. That is, there was shared variance across all items, capturing the quantity of the motivation, and unique variance (unexplained by the common core) shared across subsets of items that capture the quality of the motivation. Notably, in Sample S1A, item loadings on the global factor seemed to support the idea of SDT continuum in the sense that loadings of intrinsic items were positive and stronger (Mean λ = .69) compared to motivations that are conceptually located towards the midpoint of the continuum (identified Mean λ = .63; positive introjected Mean λ = .57). Loadings of negative introjected (Mean λ = .48) and external motivations (Mean λ = .37) were smaller, and negative for amotivation (Mean λ = -.49). This is consistent with previous applications of B-ESEM on autonomous motivations in other domains (e.g., workplace motivation; Howard et al., 2016). On the other hand, in Sample S1B, this pattern did not necessarily replicate (e.g., loadings of intrinsic items as strong as those of positive introjected).

# Table S2 *Goodness-of-Fit Statistics and Information Criteria for Models Describing the Autonomous Motivations for Romantic Pursuit (Study S1)*

|  | χ^2^ | *df* | RMSEA [90% CI] | CFI | TLI | AIC | BIC | CAIC | SABIC |
| --- | --- | --- | --- | --- | --- | --- | --- | --- | --- |
| Sample S1A (*n* = 683) | |  |  |  |  |  |  |  |  |
| CFA | 731.045 | 237 | 0.055 [0.051, 0.060] | 0.942 | 0.932 | 40852.60 | 41246.40 | 41333.40 | 40970.17 |
| ESEM | 458.756 | 147 | 0.056 [0.050, 0.062] | 0.963 | 0.931 | 40706.87 | 41508.06 | 41685.06 | 40946.06 |
| B-CFA | 939.245 | 228 | 0.068 [0.063, 0.072] | 0.916 | 0.899 | 41056.62 | 41491.16 | 41587.16 | 41186.35 |
| B-ESEM | 348.173 | 129 | 0.050 [0.044, 0.056] | 0.974 | 0.945 | 40595.45 | 41478.12 | 41673.12 | 40858.96 |
| Sample S1B (*n* = 746) | |  |  |  |  |  |  |  |  |
| CFA | 925.884 | 237 | 0.062 [0.058, 0.067] | 0.936 | 0.925 | 42806.93 | 43208.41 | 42830.20 | 42932.15 |
| ESEM | 594.199 | 147 | 0.064 [0.059, 0.069] | 0.958 | 0.922 | 42574.61 | 43391.42 | 42685.55 | 42829.37 |
| B-CFA | 1271.320 | 228 | 0.078 [0.074, 0.083] | 0.903 | 0.882 | 43127.53 | 43570.55 | 43156.23 | 43265.71 |
| B-ESEM | 343.357 | 129 | 0.047 [0.041, 0.053] | 0.980 | 0.957 | 42324.69 | 43224.56 | 42463.67 | 42605.36 |

*Note.* df = Degrees of freedom; RMSEA = Root Mean Square Error of Approximation; CI = Confidence Interval; CFI = Comparative Fit Index; TLI = Tucker-Lewis Index; AIC = Akaike Information Criterion; BIC = Bayesian Information Criterion; CAIC = Constant AIC; SABIC = Sample-size adjusted BIC; CFA = Independent cluster model–Confirmatory Factor Analysis; ESEM = Exploratory Structural Equation Modeling. Values greater than 0.90 for CFI and TLI and values smaller than 0.08 for the RMSEA were considered to support adequate fit; lower values on the AIC, CAIC, BIC, and SABIC suggest a better fit. All chi-square tests were significant at *p* < .01. Numbers are rounded to three decimal places for RMSEA, CFI, and TLI for better comparisons across the models.

# Table S3 *Standardized Factor Loadings (λ) and Uniquenesses (δ) for Confirmatory Factor Analysis and Exploratory Structural Equation Modeling for the Autonomous Motivation for Romantic Pursuit Scale (Sample S1A)*

|  | ICM-CFA | |  | ESEM | | | | | |  |
| --- | --- | --- | --- | --- | --- | --- | --- | --- | --- | --- |
| Items | λ | δ |  | F1 (λ) | F2 (λ) | F3 (λ) | F4 (λ) | F5 (λ) | F6 (λ) | δ |
| 1. Intrinsic | | |  |  |  |  |  |  |  |  |
| Item 1 | 0.86^**^ | 0.25^**^ |  | **0.94^**^** | -0.02 | -0.08^*^ | 0.05^*^ | -0.04 | 0.01 | 0.21^**^ |
| Item 2 | 0.88^**^ | 0.23^**^ |  | **0.89^**^** | -0.03 | 0.02 | 0.01 | -0.03 | -0.02 | 0.21^**^ |
| Item 3 | 0.66^**^ | 0.57^**^ |  | **0.46^**^** | 0.18^*^ | 0.07 | -0.06 | 0.09^*^ | 0.00 | 0.58^**^ |
| Item 4 | 0.68^**^ | 0.53^**^ |  | **0.51^**^** | 0.09 | 0.08 | -0.03 | 0.07 | -0.06 | 0.55^**^ |
| 2. Identified | |  |  |  |  |  |  |  |  |  |
| Item 1 | 0.85^**^ | 0.28^**^ |  | 0.10^*^ | **0.76^**^** | -0.03 | 0.02 | 0.04 | -0.02 | 0.27^**^ |
| Item 2 | 0.84^**^ | 0.28^**^ |  | -0.02 | **0.78^**^** | 0.03 | 0.10^**^ | -0.01 | -0.06 | 0.26^**^ |
| Item 3 | 0.49^**^ | 0.76^**^ |  | 0.06 | **0.46^**^** | 0.07 | -0.11^*^ | -0.04 | 0.02 | 0.75^**^ |
| Item 4 | 0.84^**^ | 0.30^**^ |  | 0.08 | **0.67^**^** | 0.08^*^ | 0.01 | -0.02 | -0.10^**^ | 0.32^**^ |
| 3. Positive introjected | | |  |  |  |  |  |  |  |  |
| Item 1 | 0.66^**^ | 0.56^**^ |  | 0.05 | 0.21^**^ | **0.46^**^** | 0.02 | 0.10^*^ | 0.03 | 0.55^**^ |
| Item 2 | 0.76^**^ | 0.43^**^ |  | -0.12^**^ | 0.07 | **0.73^**^** | 0.06 | 0.02 | 0.02 | 0.42^**^ |
| Item 3 | 0.83^**^ | 0.30^**^ |  | 0.06 | -0.14^**^ | **0.87^**^** | -0.01 | 0.00 | -0.10^**^ | 0.26^**^ |
| Item 4 | 0.84^**^ | 0.29^**^ |  | 0.03 | 0.02 | **0.85^**^** | 0.02 | -0.03 | 0.06^*^ | 0.28^**^ |
| 4. Negative introjected | | | |  |  |  |  |  |  |  |
| Item 1 | 0.87^**^ | 0.24^**^ |  | 0.06^*^ | -0.05 | 0.07^*^ | **0.84^**^** | -0.04 | -0.04 | 0.24^**^ |
| Item 2 | 0.88^**^ | 0.22^**^ |  | 0.00 | -0.03 | 0.07 | **0.86^**^** | 0.01 | 0.03 | 0.22^**^ |
| Item 3 | 0.91^**^ | 0.18^**^ |  | -0.02 | 0.07^*^ | -0.11^**^ | **0.95^**^** | 0.01 | -0.01 | 0.15^**^ |
| Item 4 | 0.88^**^ | 0.22^**^ |  | -0.02 | 0.02 | 0.05 | **0.81^**^** | 0.06^*^ | -0.01 | 0.22^**^ |
| 5. External | | |  |  |  |  |  |  |  |  |
| Item 1 | 0.78^**^ | 0.39^**^ |  | 0.10^*^ | -0.11^*^ | 0.04 | 0.04 | **0.75^**^** | 0.02 | 0.38^**^ |
| Item 2 | 0.72^**^ | 0.48^**^ |  | 0.01 | 0.06 | -0.02 | 0.03 | **0.69^**^** | 0.07 | 0.50^**^ |
| Item 3 | 0.79^**^ | 0.37^**^ |  | -0.04 | -0.08 | 0.04 | -0.10^**^ | **0.87^**^** | -0.10^**^ | 0.31^**^ |
| Item 4 | 0.75^**^ | 0.43^**^ |  | -0.06 | 0.11^*^ | -0.06 | 0.08 | **0.72^**^** | 0.01 | 0.45^**^ |
| 6. Amotivation | | |  |  |  |  |  |  |  |  |
| Item 1 | 0.74^**^ | 0.46^**^ |  | -0.10 | 0.03 | -0.06 | -0.08^*^ | -0.03 | **0.64^**^** | 0.46^**^ |
| Item 2 | 0.83^**^ | 0.31^**^ |  | -0.03 | -0.07 | 0.00 | -0.01 | -0.04 | **0.74^**^** | 0.35^**^ |
| Item 3 | 0.70^**^ | 0.52^**^ |  | 0.09^*^ | -0.02 | 0.00 | 0.04 | 0.02 | **0.78^**^** | 0.47^**^ |
| Item 4 | 0.81^**^ | 0.34^**^ |  | 0.00 | -0.02 | 0.04 | 0.00 | 0.03 | **0.85^**^** | 0.29^**^ |

*Note:* Boldface indicates target ESEM factor loadings.

^*^*p* < .05, ^**^*p* < .01.

# Table S4 *Standardized Factor Loadings (λ) and Uniquenesses (δ) for Confirmatory Factor Analysis and Exploratory Structural Equation Modeling for the Autonomous Motivation for Romantic Pursuit Scale (Sample S1B)*

|  | ICM-CFA | |  | ESEM | | | | | |  |
| --- | --- | --- | --- | --- | --- | --- | --- | --- | --- | --- |
| Items | λ | δ |  | F1 (λ) | F2 (λ) | F3 (λ) | F4 (λ) | F5 (λ) | F6 (λ) | δ |
| 1. Intrinsic | | |  |  |  |  |  |  |  |  |
| Item 1 | 0.87^**^ | 0.24^**^ |  | **0.79^**^** | 0.10 | -0.09^*^ | 0.08^*^ | -0.01 | -0.07 | 0.23^**^ |
| Item 2 | 0.89^**^ | 0.20^**^ |  | **0.78^**^** | 0.04 | 0.10^*^ | 0.04 | -0.06^*^ | -0.06 | 0.21^**^ |
| Item 3 | 0.71^**^ | 0.49^**^ |  | **0.65^**^** | -0.01 | 0.10^*^ | -0.03 | 0.06 | -0.03 | 0.48^**^ |
| Item 4 | 0.78^**^ | 0.40^**^ |  | **0.71^**^** | 0.05 | 0.04 | 0.01 | 0.04 | -0.01 | 0.38^**^ |
| 2. Identified | |  |  |  |  |  |  |  |  |  |
| Item 1 | 0.86^**^ | 0.25^**^ |  | -0.02 | **0.89^**^** | 0.03 | -0.01 | 0.02 | 0.02 | 0.22^**^ |
| Item 2 | 0.85^**^ | 0.28^**^ |  | -0.14^**^ | **0.87^**^** | 0.05 | 0.08^*^ | -0.01 | -0.06 | 0.23^**^ |
| Item 3 | 0.55^**^ | 0.70^**^ |  | 0.35^**^ | **0.36^**^** | -0.06 | -0.06 | 0.06 | 0.07 | 0.65^**^ |
| Item 4 | 0.86^**^ | 0.26^**^ |  | 0.13^**^ | **0.66^**^** | 0.04 | -0.02 | -0.01 | -0.10^**^ | 0.28^**^ |
| 3. Positive introjected | | |  |  |  |  |  |  |  |  |
| Item 1 | 0.73^**^ | 0.46^**^ |  | 0.08 | 0.19^**^ | **0.58^**^** | -0.06 | 0.07^*^ | 0.01 | 0.44^**^ |
| Item 2 | 0.74^**^ | 0.45^**^ |  | -0.06 | 0.02 | **0.64^**^** | 0.10^*^ | 0.12^**^ | 0.03 | 0.43^**^ |
| Item 3 | 0.86^**^ | 0.26^**^ |  | 0.05 | -0.09 | **0.87^**^** | 0.02 | -0.03 | -0.06^*^ | 0.25^**^ |
| Item 4 | 0.88^**^ | 0.23^**^ |  | 0.00 | 0.02 | **0.90^**^** | 0.01 | -0.05^*^ | 0.00 | 0.21^**^ |
| 4. Negative introjected | | | |  |  |  |  |  |  |  |
| Item 1 | 0.91^**^ | 0.18^**^ |  | -0.01 | 0.00 | -0.03 | **0.89^**^** | 0.02 | -0.06^*^ | 0.18^**^ |
| Item 2 | 0.92^**^ | 0.16^**^ |  | 0.06^*^ | -0.08^*^ | 0.04 | **0.90^**^** | 0.01 | -0.01 | 0.16^**^ |
| Item 3 | 0.93^**^ | 0.14^**^ |  | 0.03 | 0.04 | -0.05^*^ | **0.94^**^** | 0.01 | 0.02 | 0.13^**^ |
| Item 4 | 0.89^**^ | 0.20^**^ |  | -0.01 | 0.05 | 0.10^**^ | **0.81^**^** | 0.00 | 0.02 | 0.20^**^ |
| 5. External | | |  |  |  |  |  |  |  |  |
| Item 1 | 0.77^**^ | 0.40^**^ |  | -0.02 | -0.06 | 0.10^*^ | -0.04 | **0.79^**^** | -0.02 | 0.35^**^ |
| Item 2 | 0.75^**^ | 0.44^**^ |  | -0.03 | 0.07 | -0.05 | 0.09 | **0.66^**^** | 0.01 | 0.50^**^ |
| Item 3 | 0.82^**^ | 0.33^**^ |  | 0.03 | -0.06 | 0.01 | -0.07^*^ | **0.90^**^** | -0.06 | 0.24^**^ |
| Item 4 | 0.74^**^ | 0.46^**^ |  | -0.02 | 0.09 | -0.05 | 0.09 | **0.65^**^** | 0.06 | 0.52^**^ |
| 6. Amotivation | | |  |  |  |  |  |  |  |  |
| Item 1 | 0.78^**^ | 0.39^**^ |  | -0.05 | 0.05 | -0.02 | -0.11^**^ | -0.05 | **0.72^**^** | 0.38^**^ |
| Item 2 | 0.85^**^ | 0.27^**^ |  | 0.04 | -0.09^*^ | -0.03 | -0.03 | 0.01 | **0.79^**^** | 0.29^**^ |
| Item 3 | 0.71^**^ | 0.50^**^ |  | 0.04 | -0.03 | 0.08^*^ | 0.06^*^ | 0.00 | **0.79^**^** | 0.45^**^ |
| Item 4 | 0.86^**^ | 0.27^**^ |  | -0.07 | 0.02 | -0.04 | 0.05 | 0.00 | **0.84^**^** | 0.25^**^ |

*Note:* Boldface indicates target ESEM factor loadings.

^*^*p* < .05, ^**^*p* < .01.

# Table S5 *Standardized Factor Loadings (λ) and Uniquenesses (δ) for Bifactor Exploratory Structural Equation Modeling (B-ESEM) for the Autonomous Motivation for Romantic Pursuit Scale (Sample S1A)*

|  | Global  Factor (λ) | Specific  Factors (λ) | | | | | | |
| --- | --- | --- | --- | --- | --- | --- | --- | --- |
| Items | G | S1 | S2 | S3 | S4 | S5 | S6 | δ |
| 1. Intrinsic |  |  |  |  |  |  |  |  |
| Because I enjoy being in a relationship. | 0.63^**^ | **0.54^*^** | 0.12^*^ | -0.08 | -0.13^**^ | -0.10^*^ | -0.06 | 0.26 |
| Because I feel good being in a relationship. | 0.64^**^ | **0.63^**^** | 0.10 | 0.00 | -0.12^**^ | -0.07 | -0.08 | 0.14 |
| Because being in a relationship is interesting. | 0.71^**^ | **0.07** | -0.07 | -0.13 | -0.20^**^ | -0.06 | 0.10 | 0.41^**^ |
| Because being in a relationship is fun. | 0.77^**^ | **0.08** | -0.18^*^ | -0.16^*^ | -0.20^**^ | -0.10 | 0.08 | 0.28 |
| ω |  | .62 |  |  |  |  |  |  |
| 2. Identified |  |  |  |  |  |  |  |  |
| Because I strongly value being in a relationship. | 0.71^**^ | 0.10^*^ | **0.45^**^** | -0.05 | -0.04 | -0.03 | -0.05^*^ | 0.28^**^ |
| Because being in a relationship is personally important to me. | 0.69^**^ | 0.05 | **0.51^**^** | 0.02 | 0.07^*^ | -0.04 | -0.09^**^ | 0.24^**^ |
| Because it is my personal choice to be in a relationship. | 0.39^**^ | 0.05 | **0.27^**^** | 0.01 | -0.12^**^ | -0.08 | -0.01 | 0.75^**^ |
| Because being in a relationship is meaningful to me. | 0.71^**^ | 0.07 | **0.38^**^** | 0.01 | -0.04 | -0.08^**^ | -0.10^**^ | 0.32^**^ |
| ω |  |  | .62 |  |  |  |  |  |
| 3. Positive introjected |  |  |  |  |  |  |  |  |
| Because I would feel proud of being in a relationship. | 0.59^**^ | -0.01 | 0.08^*^ | **0.29^**^** | 0.08^*^ | 0.11^**^ | 0.05 | 0.55^**^ |
| Because I want to prove to myself that I am capable of being in a relationship. | 0.52^**^ | -0.13^**^ | 0.00 | **0.49^**^** | 0.20^**^ | 0.12^**^ | 0.05 | 0.42^**^ |
| Because being in a relationship boosts my self-esteem. | 0.60^**^ | 0.00 | -0.08^**^ | **0.58^**^** | 0.14^**^ | 0.10^**^ | -0.05 | 0.26^**^ |
| Because I want to feel good about myself by being in a relationship. | 0.58^**^ | 0.00 | 0.03 | **0.59^**^** | 0.16^**^ | 0.09^**^ | 0.06^*^ | 0.28^**^ |
| ω |  |  |  | .72 |  |  |  |  |
| 4. Negative introjected |  |  |  |  |  |  |  |  |
| Because not being in a relationship would make me feel like a bit of a loser. | 0.50^**^ | -0.03 | -0.03 | 0.12^**^ | **0.70^**^** | 0.07^*^ | -0.02 | 0.24^**^ |
| Because not being in a relationship would make me feel like there's something wrong with me. | 0.46^**^ | -0.08^*^ | -0.05 | 0.13^**^ | **0.73^**^** | 0.12^**^ | 0.04 | 0.22^**^ |
| Because I would feel like a failure if I wasn't in a relationship. | 0.47^**^ | -0.07^*^ | 0.02 | 0.02 | **0.78^**^** | 0.11^**^ | 0.00 | 0.15^**^ |
| Because I don't want to feel bad about myself by not being in a relationship. | 0.48^**^ | -0.06 | 0.01 | 0.14^**^ | **0.70^**^** | 0.17^**^ | -0.01 | 0.22^**^ |
| ω |  |  |  |  | .91 |  |  |  |
| 5. External |  |  |  |  |  |  |  |  |
| Because it would make the people close to me happy if I were in a relationship. | 0.38^**^ | 0.02 | -0.07 | 0.12^**^ | 0.16^**^ | **0.66^**^** | 0.06 | 0.38^**^ |
| Because it would be easier to maintain my social connections if I were in a relationship. | 0.35^**^ | -0.05 | 0.00 | 0.05 | 0.12^**^ | **0.59^**^** | 0.11^**^ | 0.50^**^ |
| Because the people I value would like to see me in a relationship. | 0.37^**^ | -0.07 | -0.09^*^ | 0.10^*^ | 0.07 | **0.73^**^** | -0.01 | 0.31^**^ |
| Because being in a relationship would make my relationships with my friends easier. | 0.37^**^ | -0.07 | 0.04 | 0.04 | 0.18^**^ | **0.62^**^** | 0.05 | 0.44^**^ |
| ω |  |  |  |  |  | .81 |  |  |
| 6. Amotivation |  |  |  |  |  |  |  |  |
| There is no reason I want to be in a relationship. | -0.51^**^ | -0.08 | -0.04 | -0.04 | -0.06^*^ | 0.00 | **0.51^**^** | 0.46^**^ |
| There is nothing that really motivates me to be in a relationship. | -0.55^**^ | -0.06 | -0.08^*^ | 0.02 | 0.01 | 0.03 | **0.58^**^** | 0.35^**^ |
| There may be good reasons to be in a relationship, but personally I’m not sure what they are. | -0.36^**^ | 0.00 | -0.07^*^ | 0.00 | 0.02 | 0.06 | **0.63^**^** | 0.46^**^ |
| I can’t think of anything that makes a relationship something I aspire to. | -0.52^**^ | 0.00 | 0.00 | 0.08^*^ | 0.04 | 0.11^**^ | **0.66^**^** | 0.28^**^ |
| ω | .91 |  |  |  |  |  | .79 |  |

*Note:* Boldface indicates targeted S loadings. Omega coefficients (ω) were computed based on McDonald’s (1970) formula.

^*^*p* < .05**.** ^**^*p* < .01

# Table S6 *Standardized Factor Loadings (λ) and Uniquenesses (δ) for Bifactor Exploratory Structural Equation Modeling (B-ESEM) for the Autonomous Motivation for Romantic Pursuit Scale (Sample S1B)*

|  | Global  Factor | Specific  Factors | | | | | | |
| --- | --- | --- | --- | --- | --- | --- | --- | --- |
| Items | G (λ) | S1 (λ) | S2 (λ) | S3 (λ) | S4 (λ) | S5 (λ) | S6 (λ) | δ |
| 1. Intrinsic |  |  |  |  |  |  |  |  |
| Because I enjoy being in a relationship. | 0.64^**^ | **0.56^**^** | 0.13^**^ | -0.08^**^ | -0.10^**^ | -0.07^*^ | -0.12^**^ | 0.22^**^ |
| Because I feel good being in a relationship. | 0.68^**^ | **0.55^**^** | 0.09^**^ | 0.04 | -0.10^**^ | -0.09^**^ | -0.11^**^ | 0.20^**^ |
| Because being in a relationship is interesting. | 0.60^**^ | **0.39^**^** | -0.01 | 0.00 | -0.13^**^ | -0.04 | -0.01 | 0.47^**^ |
| Because being in a relationship is fun. | 0.64^**^ | **0.44^**^** | 0.03 | -0.04 | -0.14^**^ | -0.07^**^ | -0.01 | 0.38^**^ |
| ω |  | .75 |  |  |  |  |  |  |
| 2. Identified |  | | | | | | | |
| Because I strongly value being in a relationship. | 0.72^**^ | 0.09^**^ | **0.49^**^** | -0.01 | -0.06^**^ | -0.03 | -0.05^*^ | 0.22^**^ |
| Because being in a relationship is personally important to me. | 0.73^**^ | 0.00 | **0.48^**^** | 0.01 | 0.04 | -0.04 | -0.10^**^ | 0.23^**^ |
| Because it is my personal choice to be in a relationship. | 0.50^**^ | 0.21^**^ | **0.17^**^** | -0.12^**^ | -0.15^**^ | -0.06 | 0.05 | 0.64^**^ |
| Because being in a relationship is meaningful to me. | 0.73^**^ | 0.15^**^ | **0.37^**^** | -0.03 | -0.09^**^ | -0.08^**^ | -0.11^**^ | 0.28^**^ |
| ω |  |  | .62 |  |  |  |  |  |
| 3. Positive introjected |  | | | | | | | |
| Because I would feel proud of being in a relationship. | 0.68^**^ | 0.01 | 0.05 | **0.31^**^** | 0.01 | 0.04 | 0.07^*^ | 0.43^**^ |
| Because I want to prove to myself that I am capable of being in a relationship. | 0.58^**^ | -0.13^**^ | -0.06^*^ | **0.39^**^** | 0.19^**^ | 0.14^**^ | 0.11^**^ | 0.42^**^ |
| Because being in a relationship boosts my self-esteem. | 0.65^**^ | 0.01 | -0.05^*^ | **0.55^**^** | 0.15^**^ | 0.07^**^ | 0.00 | 0.24^**^ |
| Because I want to feel good about myself by being in a relationship. | 0.65^**^ | -0.01 | 0.01 | **0.59^**^** | 0.15^**^ | 0.07^**^ | 0.03 | 0.20^**^ |
| ω |  |  |  | .72 |  |  |  |  |
| 4. Negative introjected |  | | | | | | | |
| Because not being in a relationship would make me feel like a bit of a loser. | 0.52^**^ | -0.09^**^ | -0.02 | 0.06^**^ | **0.72^**^** | 0.13^**^ | -0.03 | 0.18^**^ |
| Because not being in a relationship would make me feel like there's something wrong with me. | 0.52^**^ | -0.06^**^ | -0.07^**^ | 0.10^**^ | **0.73^**^** | 0.13^**^ | 0.01 | 0.16^**^ |
| Because I would feel like a failure if I wasn't in a relationship. | 0.54^**^ | -0.07^**^ | 0.00 | 0.05^**^ | **0.75^**^** | 0.12^**^ | 0.02 | 0.13^**^ |
| Because I don't want to feel bad about myself by not being in a relationship. | 0.56^**^ | -0.09^**^ | 0.00 | 0.13^**^ | **0.67^**^** | 0.12^**^ | 0.03 | 0.20^**^ |
| ω |  |  |  |  | .92 |  |  |  |
| 5. External |  |  |  |  |  |  |  |  |
| There is no reason I want to be in a relationship. | 0.51^**^ | -0.22^**^ | -0.24^**^ | 0.00 | 0.06 | **0.52^**^** | 0.20^**^ | 0.33^**^ |
| There is nothing that really motivates me to be in a relationship. | 0.29^**^ | 0.05 | 0.11^*^ | 0.12^**^ | 0.21^**^ | **0.74^**^** | -0.03 | 0.29^**^ |
| There may be good reasons to be in a relationship, but personally I’m not sure what they are. | 0.56^**^ | -0.20^**^ | -0.24^**^ | -0.07 | 0.02 | **0.60^**^** | 0.17^**^ | 0.19^**^ |
| I can’t think of anything that makes a relationship something I aspire to. | 0.26^**^ | 0.08^*^ | 0.15^**^ | 0.14^**^ | 0.21^**^ | **0.78^**^** | -0.02 | 0.24^*^ |
| ω |  |  |  |  |  | .87 |  |  |
| 6. Amotivation |  | | | | | | | |
| There is no reason I want to be in a relationship. | -0.60^**^ | -0.06 | 0.00 | 0.02 | -0.08^**^ | 0.02 | **0.50^**^** | 0.38^**^ |
| There is nothing that really motivates me to be in a relationship. | -0.61^**^ | -0.04 | -0.09^**^ | 0.02 | -0.02 | 0.08^**^ | **0.57^**^** | 0.29^**^ |
| There may be good reasons to be in a relationship, but personally I’m not sure what they are. | -0.43^**^ | -0.05 | -0.07^*^ | 0.09^**^ | 0.06^*^ | 0.08^**^ | **0.58^**^** | 0.45^**^ |
| I can’t think of anything that makes a relationship something I aspire to. | -0.61^**^ | -0.10^**^ | -0.03 | 0.03 | 0.06^**^ | 0.10^**^ | **0.59^**^** | 0.25^**^ |
| ω | .93 |  |  |  |  |  | .79 |  |

*Note:* Boldface indicates targeted S loadings. Omega coefficients (ω) were computed based on McDonald’s (1970) formula.

^*^*p* < .05**.** ^**^*p* < .01

# Table S7 *Correlations in Sample 1A (Study 1 in the Manuscript)*

|  | Att.Anx | Att.Avo | App.G | Avo.G | FOBS | SO | Readiness | Desire | Serious | Casual |
| --- | --- | --- | --- | --- | --- | --- | --- | --- | --- | --- |
| Intrinsic | .09 | -.21^**^ | .33^**^ | .10^*^ | .18^**^ | .11^*^ | .41^**^ | .52^**^ | .47^**^ | .18^**^ |
| Identified | .12^**^ | -.15^**^ | .28^**^ | .22^**^ | .32^**^ | -.02 | .48^**^ | .60^**^ | .58^**^ | .06 |
| Pos int. | .21^**^ | -.02 | .09^*^ | .20^**^ | .51^**^ | .02 | .25^**^ | .39^**^ | .33^**^ | .13^**^ |
| Neg. int. | .18^**^ | .09 | -.06 | .05 | .47^**^ | .06 | .10^*^ | .22^**^ | .20^**^ | .14^**^ |
| External | .12^**^ | .05 | -.11^*^ | -.03 | .31^**^ | -.00 | -.02 | .05 | .05 | .01 |
| Amotivation | -.08 | .11^*^ | -.23^**^ | -.17^**^ | -.19^**^ | -.05 | -.50^**^ | -.59^**^ | -.51^**^ | -.11^*^ |

*Notes*. Att.anx = attachment anxiety; Att.avo = attachment avoidance; App.G = approach social goals; Avo.G = avoidance social goals; FOBS = fear of being single; SO = sociosexual orientation; CS = communal strength; Readiness = commitment readiness; Desire = desire for a partner; Serious = interest in serious relationships; Casual = interest in casual relationships. **p* < .05, ***p* < .01.

# Table S8 *Correlations in Sample 1B (Study 1 in the Manuscript)*

|  | Att.Anx | Att.Avo | App.G | Avo.G | FOBS | SO | Communal | Readiness | Desire | Serious | Casual |
| --- | --- | --- | --- | --- | --- | --- | --- | --- | --- | --- | --- |
| Intrinsic | .08^*^ | -.23^**^ | .27^**^ | .04 | .17^**^ | .04 | .23^**^ | .50^**^ | .55^**^ | .59^**^ | .12^**^ |
| Identified | .10^**^ | -.17^**^ | .25^**^ | .05 | .27^**^ | -.10^**^ | .25^**^ | .48^**^ | .59^**^ | .59^**^ | .03 |
| Pos. int. | .22^**^ | -.07^*^ | .01 | .04 | .43^**^ | .08^*^ | .11^**^ | .28^**^ | .40^**^ | .34^**^ | .18^**^ |
| Neg. int. | .29^**^ | .01 | -.08^*^ | .02 | .54^**^ | .01 | .06 | .10^**^ | .24^**^ | .16^**^ | .12^**^ |
| External | .15^**^ | .03 | -.08^*^ | .05 | .35^**^ | 0.02 | .03 | .03 | .16^**^ | .10^**^ | .10^**^ |
| Amotivation | -.05 | .19^**^ | -.24^**^ | -.01 | -.21^**^ | 0.02 | -.25^**^ | -.52^**^ | -.58^**^ | -.54^**^ | -.06 |

*Notes*. Att.anx = attachment anxiety; Att.avo = attachment avoidance; App.G = approach social goals; Avo.G = avoidance social goals; FOBS = fear of being single; SO = sociosexual orientation; Communal = communal strength; Readiness = commitment readiness; Desire = desire for a partner; Serious = interest in serious relationships; Casual = interest in casual relationships. **p* < .05, ***p* < .01.

# Table S9 *Results from the Logistic Regression Models Predicting Having Entered a Relationship by Time 2 (Study 2)*

|  | **Model 1** | | | **Model 2** | | | **Model 3** | | | **Model 4** | | | **Model 5** | | | **Model 6** | | |
| --- | --- | --- | --- | --- | --- | --- | --- | --- | --- | --- | --- | --- | --- | --- | --- | --- | --- | --- |
| *Predictors* | *OR* | *z* | *p* | *OR* | *z* | *p* | *OR* | *z* | *p* | *OR* | *z* | *p* | *OR* | *z* | *p* | *OR* | *z* | *p* |
| Intrinsic | 1.59 | 7.35 | <.001 |  |  |  |  |  |  |  |  |  |  |  |  |  |  |  |
| Identified |  |  |  | 1.39 | 6.46 | <.001 |  |  |  |  |  |  |  |  |  |  |  |  |
| Pos. int. |  |  |  |  |  |  | 0.96 | -1.05 | .30 |  |  |  |  |  |  |  |  |  |
| Neg. int. |  |  |  |  |  |  |  |  |  | 0.85 | -4.10 | <.001 |  |  |  |  |  |  |
| External |  |  |  |  |  |  |  |  |  |  |  |  | 0.91 | -2.06 | .039 |  |  |  |
| Amot. |  |  |  |  |  |  |  |  |  |  |  |  |  |  |  | 0.89 | -2.74 | .006 |

*Notes*. Gender and age are included as covariates. This table is equivalent to Model 1 in Table 4 but shows results from a series of models that included only one motivation type as a predictor.

# Table S10 *Results from the Logistic Regression Models Predicting Being in a Relationship at Time 2 (Study 2)*

|  | **Model 1** | | | **Model 2** | | | **Model 3** | | |
| --- | --- | --- | --- | --- | --- | --- | --- | --- | --- |
| *Predictors* | *OR* | *z* | *p* | *OR* | *z* | *p* | *OR* | *z* | *p* |
| Sex (female) | 1.26 | 2.21 | **.03** | 1.25 | 2.16 | .03 | 1.18 | 1.50 | .13 |
| Sex (other) | 1.09 | 0.11 | .91 | 1.10 | 0.12 | .90 | 1.27 | 0.30 | .76 |
| Age | 0.98 | -2.24 | **.03** | 0.98 | -2.17 | .03 | 0.98 | -2.16 | **.03** |
| *AMRRS* |  |  |  |  |  |  |  |  |  |
| Intrinsic | 1.37 | 3.11 | **.002** | 1.36 | 3.07 | .002 | 1.24 | 2.03 | **.04** |
| Identified | 1.40 | 3.88 | **<.001** | 1.40 | 3.88 | <.001 | 1.21 | 2.07 | **.04** |
| Positive introjected | 0.93 | -0.97 | .33 | 0.93 | -0.95 | .34 | 0.94 | -0.88 | .38 |
| Negative introjected | 0.88 | -2.01 | .05 | 0.89 | -1.81 | .07 | 0.89 | -1.73 | .08 |
| External | 0.90 | -1.53 | .13 | 0.90 | -1.57 | .12 | 0.86 | -2.24 | **.03** |
| Amotivation | 1.12 | 1.72 | **.09** | 1.12 | 1.76 | .08 | 1.20 | 2.65 | **.008** |
| *Well-being* |  |  |  |  |  |  |  |  |  |
| Life satisfaction |  |  |  | 1.05 | 0.91 | .37 |  |  |  |
| *Romantic desire* |  |  |  |  |  |  |  |  |  |
| Desire for partner |  |  |  |  |  |  | 0.99 | -0.18 | .85 |
| Casual interest |  |  |  |  |  |  | 0.94 | -1.86 | .06 |
| Serious interest |  |  |  |  |  |  | 0.88 | -2.20 | **.03** |
| Intentions |  |  |  |  |  |  | 1.40 | 9.08 | **< .001** |

*Notes. N* = 3186. An Odds Ratio (OR) higher than 1 indicates that higher values of the predictor variable were associated with higher odds of currently being in a relationship at Time 2.

# Table S11 *Results from the Regression Models Predicting Relationship Quality (Study 2)*

|  | **Satisfaction** | | | **Investment** | | | **Quality of Alternatives** | | | **Commitment** | | |
| --- | --- | --- | --- | --- | --- | --- | --- | --- | --- | --- | --- | --- |
| *Predictors* | *b* | *t* | *p* | *b* | *t* | *p* | *b* | *t* | *p* | *b* | *t* | *p* |
| Intrinsic | 0.30 | 1.96 | .05 | 0.07 | 0.36 | .72 | 0.16 | 0.76 | .45 | 0.23 | 1.46 | .15 |
| Identified | -0.04 | -0.35 | .73 | 0.16 | 0.91 | .36 | 0.13 | 0.76 | .45 | 0.11 | 0.83 | .41 |
| Positive introjected | 0.06 | 0.53 | .60 | 0.27 | 1.89 | .06 | -0.09 | -0.67 | .51 | -0.01 | -0.07 | .95 |
| Negative introjected | -0.08 | -0.81 | .42 | -0.12 | -0.90 | .37 | 0.08 | 0.61 | .54 | 0.02 | 0.17 | .87 |
| External | 0.07 | 0.72 | .47 | 0.06 | 0.42 | .68 | 0.11 | 0.79 | .43 | 0.04 | 0.35 | .72 |
| Amotivation | 0.15 | 1.50 | .13 | -0.10 | -0.75 | .46 | 0.47 | 3.48 | .001 | 0.07 | 0.67 | .50 |

*Notes*. *N* = 472.

**Notes on the relationship between amotivation and sexual satisfaction (Study 2)**

We assessed sexual satisfaction using a single-item measure (“Overall, how satisfied are you with your sex life”) at baseline. This item was positively associated with amotivation at *r* = .23.
